# Three-dimensional right ventricular free-wall strain for identifying a higher Doppler-estimated PASP subgroup in high-altitude heart disease

**DOI:** 10.3389/fcvm.2026.1885692

**Published:** 2026-07-09

**Authors:** Li Cui, Xiyi Lu, Zihan Xu, Chunrui Zhou, Lei Zhong, Qingyi Luo, Qinghui Wang

**Affiliations:** 1 Department of Ultrasound, Yan’an Hospital Affiliated with Kunming Medical University, Kunming, China; 2 Key Laboratory of Cardiovascular Disease of Yunnan Province, Kunming, China; 3 Clinical Medical Research Center for Cardiovascular Disease of Yunnan Province, Kunming, China; 4 Medical School, Kunming University of Science and Technology, Kunming, China

**Keywords:** Doppler-estimated pulmonary artery systolic pressure, high-altitude heart disease, right ventricular free-wall longitudinal strain, right ventricular–pulmonary arterial coupling, septal longitudinal strain, three-dimensional echocardiography

## Abstract

**Background:**

High-altitude heart disease (HAHD) is characterized by pulmonary hypertension and progressive right ventricular dysfunction. Tricuspid annular plane systolic excursion (TAPSE) may have limited ability to characterize right ventricular impairment at higher pulmonary pressures. We aimed to determine whether three-dimensional speckle-tracking-derived right ventricular free-wall longitudinal strain, reported as an absolute magnitude (absolute RVFWLS), better identifies an operationally defined higher Doppler-estimated pulmonary artery systolic pressure (PASP) subgroup within HAHD than TAPSE, and to explore the relationships of PASP with right ventricular volumetric remodeling and strain.

**Methods:**

This retrospective study included 100 participants who underwent standardized two-dimensional and three-dimensional echocardiography: 40 high-altitude controls and 60 patients with HAHD. Patients with HAHD were categorized using an operational analytical threshold into higher-PASP (PASP ≥60 mmHg) and lower-PASP (PASP <60 mmHg) subgroups. Single-index receiver operating characteristic (ROC) curves were compared using paired DeLong tests. A logistic model evaluated absolute RVFWLS beyond TAPSE, age, and sex. Exploratory regression-based mediation-model frameworks with 5,000 bootstrap iterations examined whether right ventricular end-systolic volume (RV-ESV) statistically accounted for the associations of PASP with three-dimensional right ventricular ejection fraction (3D-EF) and absolute RVFWLS.

**Results:**

Of the 60 patients with HAHD, 26 met the operational higher-PASP threshold. Absolute RVFWLS showed better discrimination than TAPSE, with areas under the ROC curve of 0.886 and 0.647, respectively (difference, 0.238; *P* = 0.002). Adding absolute RVFWLS to TAPSE, age, and sex improved model fit (likelihood-ratio *P* < 0.001); the odds ratio per 1-percentage-point decrement in absolute RVFWLS was 1.81 (95% confidence interval, 1.29–2.54). RV-ESV statistically accounted for 45.0% (95% confidence interval, 34.8%–67.2%) of the PASP–3D-EF association, whereas it did not materially account for the PASP–absolute RVFWLS association (−1.6%; 95% confidence interval, −8.4% to 5.7%). Septal longitudinal strain was also reduced in HAHD.

**Conclusion:**

Three-dimensional absolute RVFWLS showed better discrimination than TAPSE for identifying an operationally defined higher-PASP subgroup within HAHD. The association between PASP and absolute RVFWLS was less closely related to RV-ESV than the PASP–3D-EF association. These exploratory cross-sectional findings require prospective validation against invasive hemodynamic measurements and clinical outcomes.

## 1 Introduction

High-altitude heart disease (HAHD) is the most severe manifestation of cardiovascular maladaptation to chronic hypoxia, characterized by sustained pulmonary hypertension (PH), progressive right ventricular (RV) pressure overload, and ultimately RV–pulmonary arterial (RV–PA) uncoupling (1, 2). More than 140 million people reside permanently above 2,500 m; among these, genetically and phenotypically distinct altitude populations (3–5) remain susceptible to chronic mountain sickness (CMS) or overt HAHD, with significant associated morbidity (2). Despite the clinical importance of this condition, echocardiographic approaches to characterizing pressure-related RV dysfunction within HAHD remain underdeveloped.

In susceptible individuals, concurrent polycythemia raises blood viscosity and may augment pulmonary vascular resistance, thereby compounding the hemodynamic burden imposed by hypoxic vasoconstriction alone (1, 4). Progression to overt HAHD, with sustained RV pressure overload and eventual uncoupling, carries substantial morbidity at altitudes where specialist investigation is often limited (1, 2, 4). Non-invasive echocardiographic tools capable of detecting functional deterioration in this setting may therefore be useful for research and future clinical validation.

RV–PA coupling quantifies the mechanical efficiency of the right heart by relating contractile capacity to afterload. The tricuspid annular plane systolic excursion/pulmonary artery systolic pressure (TAPSE/PASP) ratio is the most widely used echocardiographic surrogate, with values below 0.36 mm/mmHg predicting adverse outcomes in PH (6). However, TAPSE reflects only longitudinal annular displacement and is sensitive to loading conditions and annular geometry changes that accompany RV dilatation, limiting its discriminative range within a population already carrying elevated pressures (7). Three-dimensional echocardiography with speckle-tracking now permits volumetrically derived RV ejection fraction (3D-EF) (8, 9) and RV free-wall longitudinal strain (RVFWLS) (10, 11) to be measured with improved accuracy. However, the comparative performance of absolute RVFWLS and TAPSE for identifying patients with higher Doppler-estimated PASP within HAHD has not been established in altitude-specific populations, despite validation of RVFWLS in non-altitude chronic PH (10). Recent work has also highlighted that septal deformation may provide complementary information in pressure-loaded RV states because the interventricular septum participates in both RV and LV mechanics (12, 13).

Two analytical questions motivated this analysis. First, is the association between PASP elevation and RV functional indices mainly related to RV volumetric remodeling, and is absolute RVFWLS less closely associated with chamber enlargement than 3D-EF? If so, absolute RVFWLS may provide complementary echocardiographic information about RV functional impairment. Second, whether HAHD is accompanied by left ventricular (LV) or septal functional abnormalities beyond those expected from RV pressure loading alone remains important, particularly given the preserved biventricular mechanics reported in healthy altitude trekkers (13).

We therefore examined RV–PA coupling profiles across the altitude-related phenotypes included in this study, used exploratory association analysis to evaluate whether RV ESV statistically accounted for the associations between PASP and RV functional indices, and compared the ability of absolute RVFWLS and conventional echocardiographic indices to identify an operationally defined higher-PASP subgroup within HAHD. We hypothesized that absolute RVFWLS would better discriminate between the operationally defined higher- and lower-PASP subgroups within the HAHD study sample than TAPSE, and that its cross-sectional association with PASP would be less closely related to RV volumetric remodeling than the corresponding association between PASP and 3D-EF. To our knowledge, no prior study has compared three-dimensional absolute RVFWLS with conventional echocardiographic indices for this analytical task in HAHD.

## 2 Materials and methods

### 2.1 Study design and participants

This retrospective observational study analyzed de-identified clinical and echocardiographic data collected at Yan’an Hospital Affiliated with Kunming Medical University between October 2023 and June 2025. The primary study sample comprised 100 participants evaluated under a unified protocol: 40 high-altitude controls (permanent residents at ≥3,000 m for ≥5 years, without cardiopulmonary symptoms and with Doppler-estimated PASP <30 mmHg) and 60 patients with HAHD (Doppler-estimated PASP ≥30 mmHg with echocardiographic evidence of RV dilatation or dysfunction, after exclusion of alternative causes of pulmonary hypertension, including atrial or ventricular septal defects, chronic thromboembolic disease, chronic obstructive pulmonary disease [COPD], connective tissue disease, and human immunodeficiency virus [HIV]). Both groups underwent complete two-dimensional and 3D echocardiographic assessment.

A separate CMS reference group of 35 patients was derived from an independent study protocol conducted at the same altitude. CMS was defined using the Qinghai CMS score, in accordance with the international consensus statement on chronic and subacute high-altitude diseases and relevant clinical literature (2, 14). Excessive erythrocytosis was defined as a hemoglobin concentration ≥21 g/dL in men or ≥19 g/dL in women, and CMS was classified using a consensus score ≥6. Because the CMS reference group was derived from an independent protocol, it was included solely for descriptive contextualization and was not included in the primary analysis or formal statistical comparisons.

The exclusion criteria for the primary study sample were prior cardiac surgery, more than mild valvular disease, left ventricular ejection fraction (LVEF) < 40%, atrial fibrillation or frequent ventricular ectopy, pregnancy, and inadequate acoustic windows that prevented tracking of more than two RV segments. The study was approved by the Ethics Committee of Yan’an Hospital Affiliated with Kunming Medical University (approval number: 2025-086-01). The approval covered retrospective analysis of de-identified clinical and echocardiographic data collected during the study period, and the requirement for additional informed consent for this retrospective analysis was waived by the Ethics Committee.

This was an exploratory analysis of patients with HAHD, and no formal *a priori* sample-size calculation was performed. The primary analytical target was membership in an operationally defined higher-PASP subgroup, defined as Doppler-estimated PASP ≥60 mmHg (26/60 patients, 43.3%). Contemporary European Society of Cardiology/European Respiratory Society guidelines define pulmonary hypertension hemodynamically and require right heart catheterization for diagnostic confirmation (15). This clinically informed threshold was selected because PASP >60 mmHg has historically been incorporated into the EuroSCORE as a risk factor and has been associated with worse clinical outcomes in previous structural heart disease and noncardiac surgical populations (16–18). However, because these findings were derived from non-HAHD populations and right heart catheterization and clinical outcome data were unavailable in the present study, PASP ≥60 mmHg should be interpreted as an operational threshold for exploratory subgroup analysis rather than a validated HAHD-specific severity classification, prognostic cutoff, or clinical action threshold. Because the subgroup definition and candidate echocardiographic indices were derived from the same examination, the analysis evaluated subgroup discrimination rather than prediction against an independent invasive or prognostic reference standard.

### 2.2 Echocardiographic protocol

All studies were performed by experienced sonographers using a GE Vivid E95 ultrasound system (GE Healthcare, Milwaukee, WI, USA) equipped with an M5Sc 2D phased-array transducer (1.4–4.6 MHz) and a 4 V matrix-array 3D transducer (1.4–5.5 MHz), following a standardized protocol. Three to five consecutive cardiac cycles were acquired and averaged for every parameter. Standard two-dimensional parameters included RV basal diameter, TAPSE, RV S’ tissue Doppler velocity, fractional area change (FAC), and LVEF by biplane Simpson's method. Pulmonary artery systolic pressure was estimated as 4 × (peak tricuspid regurgitation [TR] velocity)^2^ plus estimated right atrial pressure (RAP) derived from inferior vena cava diameter and respiratory collapse (19). For retrospective quality control, stored continuous-wave Doppler recordings were reviewed for all participants in the primary study sample. The highest-velocity TR signal with the clearest available spectral envelope was used for PASP estimation, and measurements were averaged over at least three consecutive cardiac cycles where reviewable stored recordings were available. RAP was estimated consistently from inferior vena cava diameter and respiratory variation according to guideline-based criteria. TR signal quality, missingness, and PASP data availability are summarized in Supplementary Table S3. Three-dimensional full-volume RV datasets were acquired from an apical four-chamber window over 4–6 consecutive cardiac cycles at a volume rate of ≥ 12 volumes per second and analyzed offline on an EchoPAC 204 workstation (GE Healthcare) using the 4D Auto-RVQ module for volumetric and 3D speckle-tracking analysis. Volumetric outputs included 3D RV end-diastolic volume (EDV), ESV, ejection fraction (3D-EF), and stroke volume.

Speckle-tracking analysis yielded RVFWLS as the mean of basal, mid, and apical free-wall segments, RV global longitudinal strain (RVGLS), and LV septal longitudinal strain (Sep-LS) at basal, mid, and apical levels. RVFWLS was reported as an absolute magnitude (absolute RVFWLS); lower values indicate worse RV free-wall longitudinal function. Septal longitudinal strain was also reported as an absolute value in tables and figures; lower values indicate worse septal deformation. Three coupling ratios were derived: TAPSE/PASP, 3D-EF/PASP, and absolute RVFWLS/PASP.

### 2.3 Reproducibility

Reproducibility of the 3D speckle-tracking and real-time three-dimensional echocardiography (RT-3DE) indices was assessed in a random sample of 30 participants by two independent experienced sonographers, who repeated all measurements blinded to group assignment and to each other's readings; the intra-observer re-read was performed at least 2 weeks after the initial measurement to minimize recall bias. Intraclass correlation coefficients (ICC, two-way mixed model, absolute agreement) and 95% confidence intervals (CIs) were computed for each index, and Bland–Altman plots were generated for absolute RVFWLS (Supplementary Tables S1 and S2; Supplementary Figure S1). Both intra- and inter-observer intraclass correlation coefficients (ICCs) exceeded 0.85 for all 3D speckle-tracking strain components and all RT-3DE volumetric and functional indices (range 0.857–0.933). Intra- and inter-observer reproducibility of TR Vmax and PASP estimation was additionally assessed in a reproducibility subsample of 30 participants with reviewable stored TR envelopes. Agreement in RAP category assignment and TR envelope quality classification was also evaluated. The corresponding results are presented in Supplementary Table S3 and Supplementary Figure S3.

### 2.4 Statistical analysis

Continuous variables are presented as mean ± standard deviation (SD) or median with interquartile range, as appropriate. Between-group comparisons used the Mann–Whitney U test for continuous variables and the chi-square or Fisher's exact test for categorical variables. The CMS reference group was presented descriptively without formal hypothesis testing. The primary study sample had complete data for the main analyses; therefore, no imputation was performed.

Exploratory cross-sectional association analyses using regression-based mediation models with 5,000 parametric bootstrap iterations were performed in the primary study sample (*n* = 100) for the pathways PASP→RV-ESV→3D-EF and PASP→RV-ESV→absolute RVFWLS, with adjustment for age and sex (20). Because all variables were measured during the same examination and the assumptions required for causal mediation could not be verified, these analyses were interpreted as exploratory association patterns rather than evidence of causal mediation.

Among patients with HAHD (*n* = 60; 26 in the operationally defined higher-PASP subgroup), receiver operating characteristic (ROC) analysis evaluated each echocardiographic index for identifying subgroup membership. Areas under the ROC curve (AUCs) were reported with 1,000-bootstrap 95% confidence intervals and compared using paired DeLong tests with Holm–Bonferroni correction (21). Coupling ratios containing PASP in the denominator were excluded from ROC analysis to avoid circular predictor–outcome dependency.

The primary incremental analysis used a parsimonious nested logistic model that added absolute RVFWLS to a base model containing TAPSE, age, and sex. Models were compared using the likelihood-ratio test and Akaike information criterion (AIC), with AUCs corrected for optimism using 500 bootstrap iterations. Calibration was assessed using the Brier score and an apparent calibration plot. Because no formal *a priori* sample-size calculation was performed and only 26 higher-PASP events were available, all multivariable, integrated discrimination improvement (IDI) (22), subgroup, and reclassification analyses were considered exploratory. The fully adjusted model, IDI analysis, and subgroup analyses are reported in the Supplementary Material. A sensitivity analysis excluded three participants without reviewable stored TR envelopes.

Continuous associations between PASP and coupling indices were characterized using smoothing splines with 500-bootstrap 95% confidence bands. Analyses were performed using Python 3.11, and two-sided *P* < 0.05 was considered statistically significant. The study was reported in accordance with the Strengthening the Reporting of Observational Studies in Epidemiology (STROBE) recommendations (23). All ROC analyses were interpreted as exploratory discrimination between operationally defined PASP subgroups rather than diagnostic or prognostic analyses against an independent reference standard.

## 3 Results

### 3.1 Participant characteristics

The primary study sample consisted of 40 high-altitude controls and 60 HAHD patients (Table 1). The two groups were similar with respect to sex (60% vs. 55% male; *P* = 0.773), body mass index (BMI), heart rate, blood pressure, altitude of residence, and prevalence of smoking, hypertension, and coronary heart disease. HAHD patients were on average seven years older than controls (55.3 ± 14.6 vs. 47.9 ± 18.3 years; *P* = 0.043). Most RV structure, function, and pulmonary pressure indices differed markedly between the primary study groups (PASP 58.2 ± 11.9 vs. 22.5 ± 3.0 mmHg; TR velocity 3.26 ± 0.44 vs. 2.08 ± 0.18 m/s; main PA diameter 28.2 ± 3.6 vs. 23.0 mm; RV basal diameter 26.2 ± 2.9 vs. 21.6 ± 2.0 mm; TAPSE 15.0 vs. 25.6 mm; RV S’ 11.5 ± 2.0 vs. 15.0 [14.0–16.0] cm/s; FAC 34.0% vs. 46.5%; all *P* < 0.001). Among HAHD patients, 26 (43.3%) had Doppler-estimated PASP ≥ 60 mmHg and were included in the operationally defined higher-PASP subgroup; 27 (45.0%) reported cardiopulmonary symptoms. Among patients in the higher-PASP subgroup, 25 of 26 (96.2%) were symptomatic; conversely, only 1 of 33 asymptomatic HAHD patients had PASP ≥ 60 mmHg. The descriptive CMS reference group (*n* = 35) had a PASP of 46.4 ± 9.9 mmHg, a TAPSE of 17.7 ± 4.3 mm, and a hemoglobin concentration of 21.4 ± 1.1 g/dL. Because this group was derived from an independent study protocol, these descriptive values should not be interpreted as evidence of a continuous or longitudinal disease trajectory among the high-altitude control, CMS, and HAHD groups. Stored TR spectral envelopes were adequate for retrospective re-review in 97 of 100 participants. Original database PASP values were available for all participants. TR Vmax and PASP estimation showed excellent intra- and inter-observer reproducibility in the reproducibility subsample (Supplementary Table S3 and Supplementary Figure S3). Exclusion of the three participants without reviewable stored TR envelopes did not materially alter the primary findings (Supplementary Material).

**Table 1:** Baseline characteristics of the primary study sample and descriptive chronic mountain sickness (CMS) reference group.

Variable	HA-Ctrl (*n* = 40)	CMS (*n* = 35)	HAHD (*n* = 60)	*P* value
Demographics				
Age (years)	47.9 ± 18.3	58.0 ± 14.6	55.3 ± 14.6	0.04
Male sex, *n* (%)	24 (60.0%)	10 (28.6%)	33 (55.0%)	0.773
BMI (kg/m^2^)	23.9 [23.5–24.3]	—	23.8 [23.0–24.4]	0.71
SBP (mmHg)	117.2 ± 15.7	—	118.0 [102.5–133.5]	0.90
DBP (mmHg)	76.8 ± 9.1	—	79.5 [71.0–90.2]	0.09
HR (bpm)	74.0 ± 11.2	—	75.3 ± 9.4	0.58
Altitude (m)	3071.0 [3011.0–3225.0]	3300.0 [2500.0–3300.0]	3200.0 [3071.0–3300.0]	0.18
Smoking, *n* (%)	8 (20.0%)	—	21 (35.0%)	0.16
Hypertension, *n* (%)	8 (20.0%)	—	16 (26.7%)	0.60
CHD, *n* (%)	5 (12.5%)	—	15 (25.0%)	0.20
Hb (g/dL)	—	21.4 ± 1.1	—	—
Hct (%)	—	65.6 ± 2.8	—	—
Echocardiography				
PASP (mmHg)	22.5 ± 3.0	46.4 ± 9.9	58.2 ± 11.9	< 0.001
TR velocity (m/s)	2.1 ± 0.2	—	3.3 ± 0.4	< 0.001
Main PA diameter (mm)	23.0 [22.0–25.0]	—	28.2 ± 3.6	< 0.001
RV basal diameter (mm)	21.6 ± 2.0	—	26.2 ± 2.9	< 0.001
TAPSE (mm)	25.6 ± 2.0	17.7 ± 4.3	15.0 [14.0–17.0]	< 0.001
RV S’ (cm/s)	15.0 [14.0–16.0]	—	11.5 ± 2.0	< 0.001
FAC (%)	46.5 ± 5.6	33.9 ± 5.2	34.0 ± 6.1	< 0.001
TAPSE/PASP (mm/mmHg)	1.16 ± 0.18	0.40 ± 0.15	0.27 [0.22–0.31]	< 0.001
LVEF (%)	62.0 ± 3.0	—	61.0 [56.0–63.0]	0.01
LVEF < 55%, *n* (%)	0 (0.0%)	—	7 (11.7%)	0.04
3D Echocardiography				
3D-EF (%)	49.8 ± 4.3	—	36.9 ± 7.9	< 0.001
Absolute RVFWLS (%)	28.6 ± 2.9	—	15.7 ± 3.6	< 0.001
3D-RV EDV (mL)	72.4 ± 14.2	—	98.0 [84.8–123.2]	< 0.001
3D-RV ESV (mL)	36.7 ± 8.5	—	62.0 [47.0–78.2]	< 0.001
Absolute Sep-LS basal (%)	23.9 ± 2.2	—	16.0 [14.0–18.0]	< 0.001
Absolute Sep-LS mid (%)	26.0 [24.0–27.0]	—	17.0 [16.0–19.0]	< 0.001
Absolute Sep-LS apical (%)	26.9 ± 2.3	—	18.0 [17.0–22.2]	< 0.001
Cardiopulmonary symptoms, *n* (%)	0 (0.0%)	—	27 (45.0%)	< 0.001

Data are presented as mean ± SD, median [interquartile range], or *n* (%), as appropriate.

The CMS reference group is shown for descriptive contextualization only and was not included in formal between-group testing.

Variables not collected under the independent CMS protocol are shown as —.

*P* values compare HA-Ctrl and HAHD only; the CMS column is descriptive.

BMI, body mass index; SBP, systolic blood pressure; DBP, diastolic blood pressure; HR, heart rate; Hb, hemoglobin; Hct, hematocrit; CHD, coronary heart disease; CMS, chronic mountain sickness; FAC, fractional area change; HA-Ctrl, high-altitude control; HAHD, high-altitude heart disease; LVEF, left ventricular ejection fraction; PA, pulmonary artery; PASP, pulmonary artery systolic pressure; RV, right ventricular; RVFWLS, right ventricular free-wall longitudinal strain; Sep-LS, septal longitudinal strain; TAPSE, tricuspid annular plane systolic excursion; TR, tricuspid regurgitation; EDV, end-diastolic volume; ESV, end-systolic volume.

### 3.2 RV–PA coupling profiles across study groups

**Figure 1:**
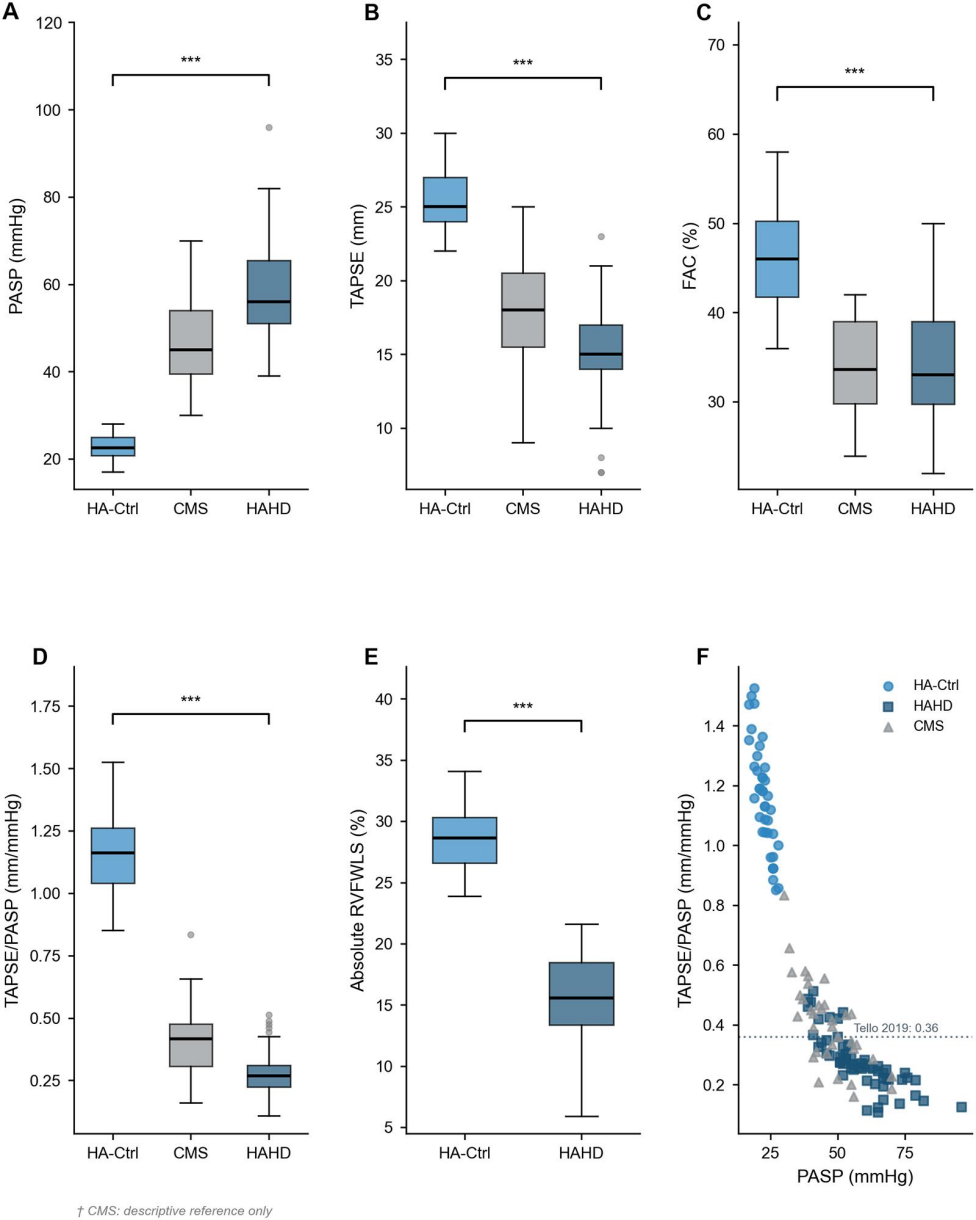
RV–PA coupling profiles across the study groups. Panels **(A–E)** show the distributions of PASP, TAPSE, FAC, TAPSE/PASP, and absolute RVFWLS, respectively, in high-altitude controls (*n* = 40) and patients with HAHD (*n* = 60). The separate CMS reference group (*n* = 35) is shown solely for descriptive contextualization and was not included in formal statistical comparisons. Its position relative to the other study groups should not be interpreted as evidence of a continuous or longitudinal disease trajectory. Panel **(F)** displays the relationship between PASP and TAPSE/PASP; the external PAH reference value reported by Tello et al. is shown for descriptive context only. ***P < 0.001. Points beyond the whiskers represent individual observations outside 1.5 × the interquartile range and were retained in the analyses. CMS, chronic mountain sickness; FAC, fractional area change; HAHD, high-altitude heart disease; PAH, pulmonary arterial hypertension; PASP, pulmonary artery systolic pressure; RV–PA, right ventricular–pulmonary arterial; RVFWLS, right ventricular free-wall longitudinal strain; TAPSE, tricuspid annular plane systolic excursion.

High-altitude controls displayed a relatively high TAPSE/PASP ratio of 1.16 ± 0.18 mm/mmHg, exceeding the reference range of approximately 0.8–1.0 mm/mmHg reported for lowland healthy subjects (6). In HAHD, the ratio was markedly lower at 0.27 [0.22–0.31] mm/mmHg (*P* < 0.001 vs. controls). The descriptive CMS reference group showed an intermediate TAPSE/PASP value of 0.40 ± 0.15 mm/mmHg between the high-altitude control and HAHD groups (Figure 1). Because the CMS reference group was derived from a separate protocol, this descriptive pattern should not be interpreted as evidence of a longitudinal disease trajectory.

Other coupling indices followed the same pattern (Table 1). Absolute RVFWLS was 28.6 ± 2.9% in controls versus 15.7 ± 3.6% in HAHD (*P* < 0.001); 3D-EF was 49.8 ± 4.3% versus 36.9 ± 7.9% (*P* < 0.001); and FAC was 46.5 ± 5.6% versus 34.0 ± 6.1% (*P* < 0.001). Descriptive comparisons of absolute RVFWLS and 3D-EF across PASP-defined tertiles are provided in Supplementary Figure S4. Because PASP was used as the grouping variable, these cross-sectional comparisons are not interpreted as evidence of a temporal severity gradient, disease progression, or a mechanistic relationship.

Segment-level analysis of the RV free wall revealed that basal, mid, and apical strain values were all comparably reduced in HAHD (reductions of 12.3, 13.2, and 11.2 percentage points respectively; all *P* < 0.001). The apex-to-base strain ratio did not differ between groups (0.91 vs. 0.89; *P* = 0.28), showing a relatively uniform pattern of reduced free-wall deformation rather than the apical-sparing pattern reported in PH.

### 3.3 Exploratory association analysis using mediation models

**Figure 2:**
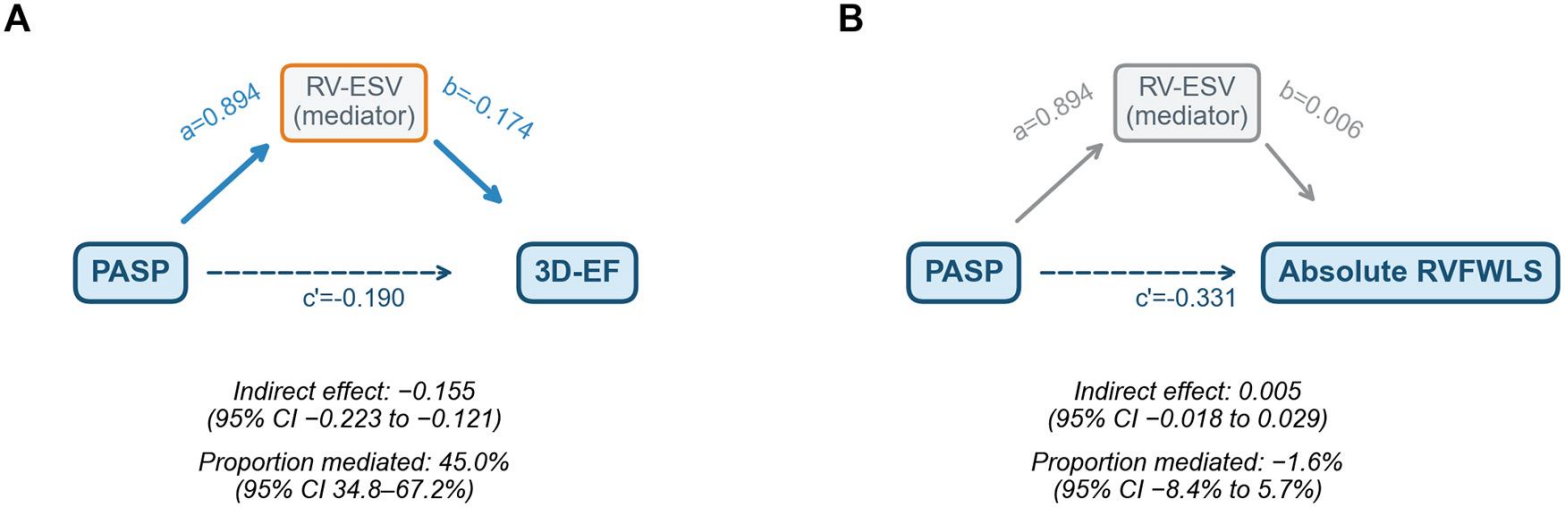
Exploratory cross-sectional association analysis using mediation-model frameworks. The two panels depict the regression-based association models examined in the primary study sample (*n* = 100). The arrows indicate the model structure and do not establish temporal or causal direction. The pathway PASP→RV-ESV→3D-EF yielded an indirect effect of −0.155 (95% CI −0.223 to −0.121; proportion mediated 45.0%, 95% CI 34.8–67.2%), whereas the pathway PASP→RV-ESV→absolute RVFWLS yielded an indirect effect of 0.005 (95% CI −0.018 to 0.029; proportion mediated −1.6%, 95% CI −8.4% to 5.7%). Because exposure, mediator, and outcome were measured cross-sectionally, these analyses are hypothesis-generating and should not be interpreted as evidence of causal mediation. ESV, end-systolic volume; PASP, pulmonary artery systolic pressure; RVFWLS, right ventricular free-wall longitudinal strain.

Two pathways were examined using exploratory regression-based mediation models in the primary study sample (*n* = 100; Figure 2). Because exposure, mediator, and outcome were measured during the same echocardiographic examination, these estimates should be interpreted as cross-sectional association patterns rather than evidence of temporal or causal pathways.

For the pathway PASP→RV-ESV→3D-EF, the indirect effect was −0.155 (95% CI −0.223 to −0.121), accounting for 45.0% (95% CI 34.8–67.2%) of the total association. This finding suggests that RV chamber volumes statistically accounted for a substantial proportion of the PASP–3D-EF association, which is expected because ejection fraction is mathematically derived from end-diastolic and ESVs.

For the pathway PASP→RV-ESV→absolute RVFWLS, the indirect effect was 0.005 (95% CI −0.018–0.029), with a mediation proportion of −1.6% (95% CI −8.4% to 5.7%); the confidence interval crossed zero. Within the limits of a cross-sectional design, RV-ESV did not materially account for the PASP–absolute RVFWLS association. This finding is compatible with the hypothesis that absolute RVFWLS is less closely related to RV volumetric remodeling than 3D-EF, but it should not be interpreted as evidence of a specific contractile mechanism. The same pattern was observed in the HAHD subgroup, although the confidence intervals were wide (mediation proportion for 3D-EF, 58.5%; 95% CI 35.3–114.1%; mediation proportion for absolute RVFWLS, 2.9%; 95% CI −8.1% to 16.5%).

### 3.4 Identification of an operationally defined higher-PASP subgroup

**Figure 3:**
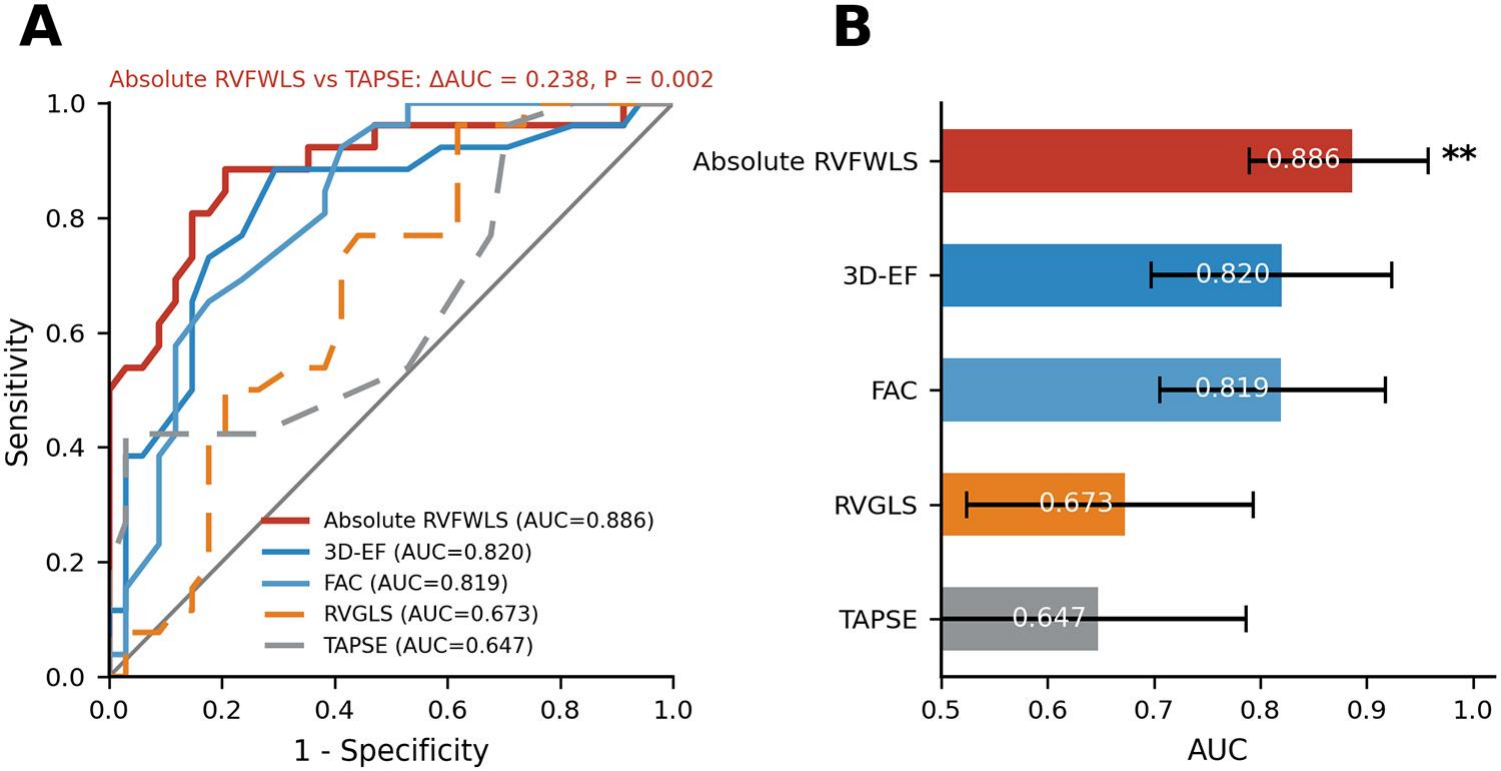
ROC-based discrimination of an operationally defined higher-PASP subgroup within HAHD. Panel **(A)** shows ROC curves for five single echocardiographic indices identifying membership in the operationally defined higher-PASP subgroup (Doppler-estimated PASP ≥ 60 mmHg; *n* = 26/60); coupling ratios containing PASP in the denominator were excluded to avoid circular predictor-outcome dependency. Panel **(B)** displays AUC estimates with bootstrap 95% confidence intervals; absolute RVFWLS (AUC 0.886) significantly outperformed TAPSE (AUC 0.647; 95% CI 0.498–0.786; *Δ*AUC 0.238, 95% CI 0.093–0.396; *P* = 0.002) by DeLong analysis. **P < 0.01. AUC, area under the curve; HAHD, high-altitude heart disease; PASP, pulmonary artery systolic pressure; RVFWLS, RV free-wall longitudinal strain.

Within HAHD (*n* = 60; 26 participants in the operationally defined higher-PASP subgroup), five echocardiographic indices were compared by ROC analysis for exploratory discrimination between the operationally defined PASP subgroups; coupling ratios containing PASP in the denominator were excluded to avoid circular predictor-outcome dependency (Table 2; Figure 3). Absolute RVFWLS achieved the highest AUC at 0.886 (95% CI 0.789–0.958). 3D-EF (AUC 0.820) and FAC (AUC 0.819) showed intermediate discrimination, whereas RVGLS (AUC 0.673) and TAPSE (AUC 0.647, 95% CI 0.498–0.786) showed lower AUCs. Absolute RVFWLS significantly outperformed TAPSE (*Δ*AUC 0.238, 95% CI 0.093–0.396; *P* = 0.002) and RVGLS (*Δ*AUC 0.213; *P* = 0.001) by paired DeLong testing. These results indicate better discrimination of an operationally defined higher-PASP subgroup within the HAHD study sample by absolute RVFWLS than by TAPSE. Prospective validation against invasive hemodynamics and clinical outcomes is required.

**Table 2:** Echocardiographic discrimination of an operationally defined higher pulmonary artery systolic pressure (PASP) subgroup within high-altitude heart disease (HAHD).

Index	AUC	95% CI	Delta AUC vs TAPSE	*P* value vs. TAPSE
Absolute RVFWLS (%)	0.886**	0.789–0.958	0.238	0.002
3D-EF (%)	0.820	0.698–0.924	0.173	–
FAC (%)	0.819	0.706–0.917	0.172	–
RVGLS (%)	0.673	0.524–0.794	0.026	–
TAPSE (mm)	0.647	0.498–0.786	Reference	Reference

AUC, area under the ROC curve; CI, confidence interval (bootstrap 1,000 iterations, seed 42). All analyses were performed within HAHD (*n* = 60). The analytical target was membership in the operationally defined higher-PASP subgroup (Doppler-estimated PASP ≥ 60 mmHg; *n* = 26), not a validated definition of severe disease, a validated risk category, a clinical action threshold, or an invasively confirmed diagnosis of severe pulmonary hypertension. Absolute RVFWLS is reported as positive absolute strain, whereas RVGLS follows the signed strain convention. *P* values are shown for exploratory pairwise DeLong comparisons reported in the main analysis; dashes indicate comparisons not reported as primary inferential tests.

** *P* < 0.01 vs. TAPSE (paired DeLong test).

In a parsimonious logistic model containing TAPSE, age and sex, adding absolute RVFWLS reduced the Akaike information criterion (AIC) by 21.7 units (from 78.7 to 57.0) and was significant by likelihood ratio test (*P* < 0.001). Because lower absolute strain reflects worse RV function, the effect is reported as the odds ratio per 1-percentage-point decrement in absolute RVFWLS, which was 1.81 (95% CI 1.29–2.54). In the sensitivity analysis excluding the three participants without reviewable stored TR envelopes, the main findings were unchanged: the HAHD analysis set contained 59 patients and 26 events, the absolute RVFWLS AUC was 0.884, the TAPSE AUC was 0.636, and the incremental logistic model remained materially identical (OR per 1-percentage-point decrement in absolute RVFWLS, 1.81; 95% CI 1.29–2.54; likelihood ratio *P* < 0.001). A fully adjusted model additionally including smoking, hypertension and coronary heart disease (events-per-variable ratio approximately 3.7) is reported in the Supplementary Material; results were directionally consistent.

Bootstrap Harrell optimism correction (500 iterations) yielded a corrected AUC of 0.892 for the TAPSE + age + sex + absolute RVFWLS model. Apparent calibration metrics (Brier score, 0.121; Cox calibration intercept, 0.000; slope, 1.001; Supplementary Figure S5) are presented descriptively, because calibration performance requires validation in an independent sample. Exploratory IDI and subgroup analyses are reported in the Supplementary Material (Supplementary Figure S2). Given the limited subgroup sizes, these analyses are exploratory, and the main inference rests on the single-index ROC comparison and the parsimonious logistic model.

### 3.5 Septal strain abnormalities

**Figure 4:**
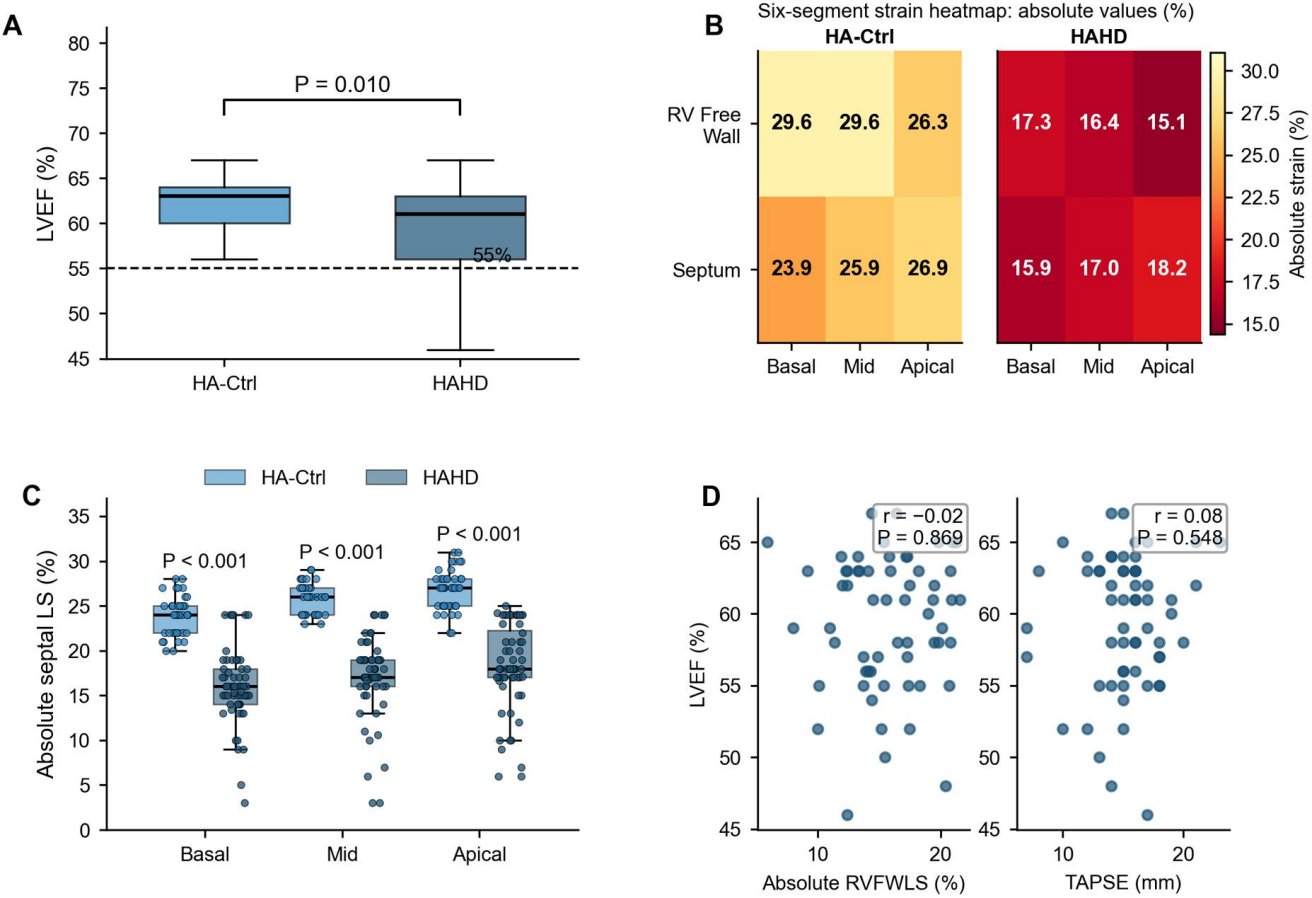
Septal strain abnormalities observed in HAHD. Panel **(A)** shows LVEF distribution in HAHD (*n* = 60) versus controls (*n* = 40); the dashed line marks 55%. Panel **(B)** shows a 6-segment regional strain heatmap comparing HA-Ctrl and HAHD; segments cover RV free wall (basal, mid, apical) and septum (basal, mid, apical), with mean absolute strain values annotated in each cell. Panel **(C)** shows absolute septal longitudinal strain at basal, mid, and apical levels (error bars, SD). Panel **(D)** shows scatter plots of LVEF against absolute RVFWLS (left) and TAPSE (right) within HAHD, with Pearson r and *P* values. HAHD, high-altitude heart disease; LVEF, left ventricular ejection fraction; LS, longitudinal strain; Sep-LS, septal longitudinal strain.

LVEF was modestly lower in HAHD than in controls. Median LVEF was 61.0 [56–63]% in HAHD versus 62.0 ± 3.0% in controls (*P* = 0.010), and 7 HAHD patients (11.7%) had LVEF below 55% compared with none of the controls. Absolute septal longitudinal strain was reduced in HAHD across all three segments: basal Sep-LS 16.0% vs. 23.9% (*Δ* −7.9 pp), mid Sep-LS 17.0% vs. 25.9% (*Δ* −8.9 pp), and apical Sep-LS 18.2% vs. 26.9% (*Δ* −8.7 pp), all *P* < 0.001 (Figures 4A–C).

Within the HAHD group, LVEF showed no significant correlation with absolute RVFWLS (r = −0.022; *P* = 0.868), 3D-EF (r = 0.117; *P* = 0.373), TAPSE (r = 0.079; *P* = 0.548), FAC (r = −0.097; *P* = 0.462), or RVGLS (r = 0.091; *P* = 0.491). The absence of meaningful RV–LV correlations suggests that the observed septal strain abnormalities may not be fully explained by conventional RV indices alone. However, these findings do not exclude ventricular interdependence, RV pressure overload, or other confounding mechanisms (Figure 4D). Sex did not modify the relationship between disease status and any echocardiographic index (all interaction *P* > 0.30); analyses were therefore not stratified by sex. A sensitivity analysis excluding the 7 HAHD patients with LVEF < 55% (*n* = 53 remaining) confirmed that septal strain impairment at all three levels persisted (all *P* < 0.001), and the absence of LVEF–RV correlations was fully preserved (LVEF–absolute RVFWLS r = 0.024, LVEF–TAPSE r = −0.039; all *P* > 0.45), indicating that the septal strain findings are not driven by overt LV dysfunction.

## 4 Discussion

High-altitude controls had a relatively high TAPSE/PASP ratio (1.16 ± 0.18 mm/mmHg), whereas HAHD patients showed marked uncoupling. Exploratory association analysis using mediation models suggested that RV-ESV did not materially account for the PASP–absolute RVFWLS association (mediation proportion −1.6%; 95% CI −8.4% to 5.7%), whereas RV volumetric remodeling statistically accounted for 45.0% (95% CI 34.8–67.2%) of the PASP–3D-EF association. Because exposure, mediator and outcome were measured at the same time, these estimates are exploratory and should not be interpreted as confirming a causal pathway. Among patients with HAHD, three-dimensional absolute RVFWLS showed better discrimination of the operationally defined higher-PASP subgroup than TAPSE (AUC 0.886 vs 0.647; *Δ*AUC 0.238; *P* = 0.002), with directionally consistent incremental-model findings. Septal strain abnormalities were also observed, but the present cross-sectional echocardiographic data cannot establish their underlying mechanisms.

The relatively high TAPSE/PASP ratio in the high-altitude control group (1.16 mm/mmHg) provides descriptive context for interpreting RV–PA coupling at altitude. The descriptive CMS reference group showed an intermediate TAPSE/PASP value, but because it was derived from a separate protocol, this pattern should not be interpreted as evidence of a longitudinal disease trajectory.

Erythrocytosis may modify high-altitude cardiopulmonary loading, but individual-level hemoglobin, hematocrit, oxygen saturation, ethnicity, and detailed residence-duration data were not comprehensively available in the primary study sample. Therefore, the present study cannot determine whether hematological or ancestry-related factors explain part of the observed RV and septal strain patterns.

The differential association patterns of absolute RVFWLS and 3D-EF should be interpreted as exploratory. The 45% volumetric mediation for 3D-EF is consistent with the dependence of ejection fraction on chamber volume, whereas the near-zero mediation proportion for absolute RVFWLS (−1.6%) is compatible with a weaker relationship to RV-ESV. These cross-sectional findings do not establish a specific contractile mechanism; they support a working hypothesis, to be tested longitudinally, that absolute RVFWLS may be less closely associated with chamber dilatation than EF-based metrics.

The AUC of 0.886 for absolute RVFWLS is consistent with values reported for 3D RVFWLS in non-altitude chronic PH study populations (10), although the present analysis addresses discrimination between the operationally defined PASP subgroups within the HAHD study sample rather than diagnosis. The superiority over TAPSE was directionally consistent across the parsimonious multivariable model, optimism correction, the exploratory IDI analysis, and the TR/PASP quality-control sensitivity analysis. Coupling ratios such as TAPSE/PASP remain useful for contextualizing RV–PA coupling across the altitude-related study groups (Figure 1F), but they were not subjected to formal ROC analysis because the analytical grouping variable was PASP-defined. Calibration findings describe internal model performance only. The analytical threshold and model require external validation against invasive hemodynamics and clinical outcomes before any clinical interpretation can be considered.

### 4.1 Comparison with previous studies

The AUC of 0.886 achieved here parallels the AUC of 0.88 reported by Vitarelli et al. for 3D RVFWLS in chronic PH study populations (10). Importantly, these analyses addressed different tasks: case-control separation in previous work versus identification of an operationally defined higher-PASP subgroup within HAHD in the present study. The retention of high discriminative performance in this more constrained setting supports the potential robustness of absolute RVFWLS as an echocardiographic marker associated with higher Doppler-estimated PASP, whereas TAPSE may be limited by its narrower dynamic range at higher pulmonary pressures.

Moceri et al. demonstrated that regional RV deformation predicted survival in PH, with more heterogeneous regional deformation patterns (24). In contrast, the present study found relatively uniform free-wall strain impairment across basal, mid, and apical RV segments, with no apex-to-base gradient. This pattern may distinguish HAHD from non-altitude PAH and suggests a more diffuse echocardiographic involvement pattern rather than a strictly regional loading-related deformation pattern.

The septal strain findings extend previous cardiovascular magnetic resonance observations in high-altitude disease by providing echocardiographic evidence of septal strain impairment at three levels (25–27). The absence of meaningful correlations between LVEF and RV indices (all |r| < 0.12) is hypothesis-generating and suggests that septal and LV functional abnormalities may not be fully explained by measured RV indices alone. However, the present cross-sectional echocardiographic data cannot determine whether these findings reflect systemic hypoxic effects, ventricular interdependence, RV pressure overload, or other unmeasured mechanisms. These findings support further evaluation of septal strain in HAHD study populations, rather than a change in clinical assessment practice at this stage.

### 4.2 Clinical implications

These findings support further evaluation of absolute RVFWLS as a candidate echocardiographic marker associated with higher Doppler-estimated PASP in HAHD. The present analysis does not establish a clinical severity threshold, a validated risk category, or a clinical action threshold. Prospective studies incorporating right heart catheterization, longitudinal outcomes, and treatment-response data are required before clinical decision-making applications can be considered.

### 4.3 Strengths

Several methodological strengths deserve acknowledgment. First, both primary study groups were recruited under a unified protocol at a single high-altitude center, reducing inter-site imaging variability and supporting direct comparability of three-dimensional speckle-tracking measurements. Second, the analytical framework combines a parsimonious exploratory subgroup-discrimination model with bootstrap optimism correction and calibration assessment, while exploratory association analysis, IDI and subgroup analyses are clearly delineated as hypothesis-generating. Third, TR/PASP image quality control and PASP reread reproducibility were assessed, and a sensitivity analysis excluding QC-flagged TR-envelope cases yielded materially unchanged results. Fourth, event counts are explicitly reported alongside events-per-variable ratios. Fifth, the independent CMS reference group provides descriptive context across the altitude-related study groups without being used for formal inference.

### 4.4 Limitations

Several limitations should be acknowledged. First, the cross-sectional design precludes temporal or causal inference. Because all mediation-model variables were measured during the same examination, the analyses cannot establish causal mediation. Furthermore, as 3D-EF is mathematically derived from RV volumes, the estimated indirect association through RV-ESV may partly reflect mathematical coupling. These findings should therefore be considered hypothesis-generating. Second, right heart catheterization data were unavailable. PASP ≥60 mmHg was used solely as an operational threshold to identify a higher-PASP subgroup and should not be interpreted as a validated severity classification, risk category, or clinical action threshold. Because the subgroup and candidate indices were derived from the same echocardiographic examination, incorporation bias may have contributed to the observed discrimination. Third, stored TR envelopes were unavailable or insufficient for retrospective review in three participants, although sensitivity analyses excluding them yielded similar results. Important high-altitude-related variables, including oxygen saturation, hematological measures, altitude-exposure duration, ethnicity, and genetic background, were also incompletely available; residual confounding therefore cannot be excluded. Fourth, the CMS reference group was derived from a separate study protocol and provided descriptive context only. Fifth, no formal *a priori* sample-size calculation was performed, and the limited sample size and event count restrict model stability. The multivariable, IDI, and subgroup analyses should therefore be considered exploratory and require external validation. Finally, the single-center design limits generalizability. Prospective multicenter studies incorporating invasive hemodynamics, high-altitude-related covariates, longitudinal outcomes, and external validation are required before clinical application can be considered.

### 4.5 Future directions

Several directions follow from the present findings. First, the association between absolute RVFWLS and higher Doppler-estimated PASP requires external validation in independent high-altitude study populations with prospective outcomes and invasive hemodynamic assessment. Second, prospective comparison of two-dimensional and three-dimensional longitudinal strain would clarify whether 3D acquisition provides incremental value in resource-limited altitude settings. Finally, multicenter studies across diverse mountain populations are needed to assess environmental, hematological, and genetic modifiers of RV–PA coupling in HAHD.

## 5 Conclusion

Within this cross-sectional high-altitude study sample, three-dimensional absolute RVFWLS showed better discrimination than conventional TAPSE for identifying an operationally defined higher-PASP subgroup. Exploratory association analysis using mediation models suggested that the PASP–absolute RVFWLS association was less closely related to RV volumetric remodeling than the PASP–3D-EF association; this should be interpreted as hypothesis-generating rather than evidence of a causal mechanism. Septal strain abnormalities were also observed, but their underlying mechanisms remain uncertain. Prospective studies with invasive hemodynamic assessment, external replication, and clinical outcomes are required before any clinical application can be considered.

## Data Availability

The raw data supporting the conclusions of this article will be made available by the authors, without undue reservation.
