# Supplementary material for: Three-dimensional right ventricular free-wall strain for identifying a higher Doppler-estimated PASP subgroup in high-altitude heart disease
Source: Front Cardiovasc Med. 2026 Jul 9;13:1885692. doi: 10.3389/fcvm.2026.1885692 (PMC13394275; doi:10.3389/fcvm.2026.1885692)
Supplement: Supplementary file 1 [file Datasheet1.docx]

# Supplementary Material

This supplementary file accompanies the revised manuscript.

The revised supplement includes Sections S1-S6, Supplementary Tables S1-S3, and Supplementary Figures S1-S5.

## S1. Fully adjusted multivariable logistic model

In addition to the parsimonious primary model (tricuspid annular plane systolic excursion [TAPSE], age, sex, and absolute right ventricular free-wall longitudinal strain [absolute RVFWLS]) reported in the main text, a fully adjusted nested logistic model added smoking, hypertension and coronary heart disease to the base set. With 26 participants in the operationally defined higher pulmonary artery systolic pressure (PASP) subgroup within the high-altitude heart disease (HAHD) group and seven candidate predictors, the events-per-variable ratio is approximately 3.7, well below the conventional ≥10 rule of thumb. The results are therefore reported here as exploratory and require external validation.

Adding absolute RVFWLS to the fully adjusted base model reduced the Akaike information criterion (AIC) by 9.2 units (from 69.08 to 59.88; likelihood ratio P < 0.001). Because lower absolute strain reflects worse right ventricular (RV) function, effects are reported as odds ratios per 1-percentage-point decrement in absolute RVFWLS: in this model, the odds ratio (OR) was 1.60 (95% confidence interval [CI] 1.14–2.24), compared with OR = 1.81 (95% CI 1.29–2.54) in the parsimonious model in the main text. The attenuation when smoking, hypertension and coronary heart disease were included is consistent with the modest events-per-variable ratio. Adding three-dimensional right ventricular ejection fraction (3D-EF) instead of absolute RVFWLS produced a smaller reduction (ΔAIC −4.84; P = 0.009); adding right ventricular global longitudinal strain (RVGLS) did not improve the model (ΔAIC +0.30; P = 0.19).

## S2. Exploratory integrated discrimination improvement analysis

The base model for the exploratory incremental-discrimination analysis was the parsimonious model containing TAPSE, age and sex. Addition of absolute RVFWLS to this base model yielded an Integrated Discrimination Improvement (IDI) of 0.20 (95% CI 0.11–0.29), computed according to Pencina et al. The primary inference, as stated in the main text, rests on the single-index receiver operating characteristic (ROC) comparison and the parsimonious logistic model.

## S3. Subgroup analyses for absolute RVFWLS versus TAPSE

Areas under the ROC curve (AUCs) for absolute RVFWLS and TAPSE for the operationally defined higher-PASP subgroup were computed within exploratory subgroups (sex, age above or below the study-sample median, smoking status, hypertension status), with 95% confidence intervals from 500 bootstrap resamples. Subgroups containing fewer than three events or three non-events were not analyzed. The forest plot in Supplementary Figure S2 shows directionally consistent superiority of absolute RVFWLS over TAPSE across all evaluable strata; the within-subgroup confidence intervals are wide and overlap appreciably given the small event counts in each stratum. These results should therefore be read as descriptive consistency rather than as evidence of effect modification.

**S3a. Descriptive PASP-tertile comparisons**

Absolute RVFWLS and 3D-EF were summarized descriptively across PASP-defined tertiles within HAHD (Supplementary Figure S4). PASP was used as the grouping variable; therefore, these cross-sectional comparisons are presented for descriptive context only and should not be interpreted as evidence of a temporal severity gradient, disease progression, or a mechanistic relationship.

## S4. Intra- and inter-observer reproducibility

Reproducibility was assessed in a randomly selected subsample of 30 participants drawn from the primary study sample (60 HAHD patients and 40 high-altitude controls), using simple random sampling without replacement. Two experienced echocardiographers, both certified in adult transthoracic imaging and 3D speckle-tracking analysis, performed all measurements independently and were blinded to clinical group, to each other, and to the original measurements made for the primary analysis. The intra-observer reliability was assessed by repeat reading of the same 30 datasets by the first reader after an interval of at least 2 weeks. Inter-observer reliability compared the first reading of reader 1 with an independent reading by reader 2.

Intraclass correlation coefficients (ICC) were calculated using a two-way mixed-effects model for absolute agreement at the single-measurement level. 95% confidence intervals were derived from the F-distribution. Bland–Altman analysis for the principal index absolute RVFWLS yielded a mean difference of −0.09 with 95% limits of agreement of −2.08 to +1.89 for the intra-observer comparison, and a mean difference of 0.09 with 95% limits of agreement of −2.64 to +2.82 for the inter-observer comparison (Supplementary Figure S1).

Supplementary Table S1. Intra- and inter-observer reproducibility of 3D speckle-tracking strain indices (n = 30).

| **Index** | **Intra-observer ICC** | **95% CI** | **Inter-observer ICC** | **95% CI** |
| --- | --- | --- | --- | --- |
| RVGLS (%) | 0.904 | 0.842–0.973 | 0.905 | 0.775–0.961 |
| absolute RVFWLS (%) | 0.927 | 0.889–0.981 | 0.912 | 0.792–0.964 |
| RVFWS-BS (%) | 0.900 | 0.829–0.989 | 0.929 | 0.830–0.971 |
| RVFWS-MS (%) | 0.923 | 0.819–0.969 | 0.895 | 0.755–0.957 |
| RVFWS-AS (%) | 0.922 | 0.857–0.976 | 0.927 | 0.824–0.970 |
| Sep-LS-BS (%) | 0.868 | 0.811–0.925 | 0.857 | 0.786–0.943 |
| Sep-LS-MS (%) | 0.901 | 0.849–0.974 | 0.894 | 0.716–0.950 |
| Sep-LS-AS (%) | 0.873 | 0.739–0.962 | 0.882 | 0.792–0.945 |

ICC computed using a two-way mixed-effects model for absolute agreement at the single-measurement level. RVGLS, RV global longitudinal strain; RVFWLS, RV free-wall longitudinal strain; RVFWS-BS/MS/AS, RV free-wall basal/mid/apical segmental strain; Sep-LS-BS/MS/AS, septal basal/mid/apical longitudinal strain.

Supplementary Table S2. Intra- and inter-observer reproducibility of RT-3DE volumetric and functional indices (n = 30).

| **Index** | **Intra-observer ICC** | **95% CI** | **Inter-observer ICC** | **95% CI** |
| --- | --- | --- | --- | --- |
| 3D-RVEDV (mL) | 0.876 | 0.781–0.941 | 0.894 | 0.737–0.933 |
| 3D-RVESV (mL) | 0.858 | 0.743–0.934 | 0.862 | 0.784–0.943 |
| 3D-RVEF (%) | 0.921 | 0.744–0.955 | 0.904 | 0.650–0.936 |
| 3D-RVSV (mL) | 0.913 | 0.794–0.965 | 0.933 | 0.864–0.959 |
| 3D-TAPSE (mm) | 0.921 | 0.811–0.968 | 0.867 | 0.806–0.947 |
| 3D-RVFAC (%) | 0.909 | 0.769–0.964 | 0.912 | 0.769–0.960 |

ICC computed using a two-way mixed-effects model for absolute agreement at the single-measurement level. 3D-RVEDV, 3D RV end-diastolic volume; 3D-RVESV, 3D RV end-systolic volume; 3D-RVEF, 3D RV ejection fraction; 3D-RVSV, 3D RV stroke volume; 3D-TAPSE, 3D-derived tricuspid annular plane systolic excursion; 3D-RVFAC, 3D RV fractional area change.

All intraclass correlation coefficients exceeded 0.85 for both the intra- and inter-observer comparisons (range 0.857–0.933 across all indices), indicating good to excellent reproducibility for both 3D speckle-tracking strain and real-time three-dimensional echocardiography (RT-3DE) volumetric measurements in this study sample. These ICC values are consistent with previously published reproducibility data for 3D speckle-tracking in chronic pulmonary hypertension cohorts (RVFWLS ICC 0.87–0.89; 3D-EF ICC 0.91–0.92; Vitarelli et al., reference 10 in the main text).

Additional tricuspid regurgitation (TR)/PASP quality control was performed to assess TR spectral quality, PASP estimation consistency, right atrial pressure (RAP) estimation consistency, and missing-data handling. The 100-participant primary study-sample quality-control review and 30-participant reproducibility analysis are summarized in Supplementary Table S3; PASP agreement is displayed in Supplementary Figure S3. The three cases without reviewable stored TR spectral envelopes are described transparently and evaluated in the sensitivity analysis below.

Supplementary Table S3. TR/PASP quality control and reproducibility.

Panel A. Retrospective quality-control review in the primary study sample (n = 100).

| Quality-control item | n (%) |
| --- | --- |
| Measurable stored TR spectral envelope available for retrospective re-review | 97/100 (97.0%) |
| Adequate stored TR spectral envelope available for retrospective re-review | 97/100 (97.0%) |
| Highest-velocity signal selected across available windows | 97/100 (97.0%) |
| Protocol-specified averaging over ≥ 3 cardiac cycles | 100/100 (100.0%) |
| IVC diameter and respiratory variation available for RAP estimation | 100/100 (100.0%) |
| Complete PASP data documented in the original database | 100/100 (100.0%) |
| Stored TR spectral envelopes unavailable or insufficient for retrospective re-review, with PASP values documented in the original database | 3/100 (3.0%) |
| Excluded from primary analysis after QC review | 0/100 (0.0%) |

Note. Three participants had PASP values documented at the original examination, but their stored TR spectral envelopes were unavailable or insufficient for retrospective re-review. Sensitivity analyses excluding these participants are reported in Section S5.

Panel B. Intra- and inter-observer reproducibility in the reproducibility subsample.

| **Parameter** | **Intra-observer ICC** | **95% CI** | **Inter-observer ICC** | **95% CI** | **Bias/95% LoA** |
| --- | --- | --- | --- | --- | --- |
| TR Vmax (m/s) | 0.995 | 0.991–0.997 | 0.994 | 0.990–0.996 | Intra: −0.01 (−0.14, 0.12);  Inter: −0.04 (−0.17, 0.09) |
| PASP (mmHg) | 0.996 | 0.992–0.998 | 0.995 | 0.992–0.997 | Intra: −0.30 (−3.72, 3.13);  Inter: −1.04 (−4.19, 2.11) |

Categorical agreement for TR envelope quality and RAP category.

| **Categorical item** | **Intra-observer agreement** | **Intra κ** | **Inter-observer agreement** | **Inter κ** |
| --- | --- | --- | --- | --- |
| TR envelope quality | 100.0% | 1.000 | 100.0% | 1.000 |
| RAP category | 100.0% | 1.000 | 100.0% | 1.000 |

ICCs were calculated using a two-way mixed-effects, absolute-agreement, single-measurement model. Bland–Altman limits of agreement were defined as mean difference ± 1.96 SD. Categorical agreement was summarized using percent agreement and kappa statistics; kappa was reported as not estimable when all observations fell within a single category. In this reproducibility subsample, both TR envelope quality and RAP category showed category variation, so kappa was estimable.

## S5. Sensitivity analyses

(a) Exclusion of patients with overt LV dysfunction. Excluding the seven HAHD patients with LVEF < 55% (n = 53 remaining) preserved the magnitude and statistical significance of septal strain impairment at all three levels (all P < 0.001) and the absence of LVEF–RV correlations (LVEF–absolute RVFWLS: r = 0.024; LVEF–TAPSE: r = −0.039; all P > 0.45), indicating that the septal strain findings are not driven by the small subgroup with overt LV dysfunction.

(b) Sensitivity analysis excluding cases without reviewable stored TR envelopes. Three participants had complete PASP values documented in the original database but lacked stored TR spectral envelopes of sufficient quality for retrospective re-review. The primary analyses were repeated after excluding these cases. The results were materially unchanged: in the HAHD subgroup, 59 patients and 26 events remained; absolute RVFWLS AUC was 0.884, TAPSE AUC was 0.636, 3D-EF AUC was 0.815, and FAC AUC was 0.814. The parsimonious incremental logistic model remained consistent (OR per 1-percentage-point decrement in absolute RVFWLS, 1.81; 95% CI 1.29–2.54; likelihood ratio P < 0.001).

## S6. Software and computational details

All statistical analyses were performed in Python 3.11 using the SciPy 1.11, NumPy 1.26, scikit-learn 1.3, statsmodels 0.14, pingouin 0.5, and matplotlib 3.8 packages. Reclassification metrics (IDI) were computed using bespoke Python implementations of the formulas of Pencina et al. Bootstrap optimism correction used 500 resamples; bootstrap AUC confidence intervals used 1,000 resamples; bootstrap mediation analysis used 5,000 parametric resamples. Two-sided P < 0.05 was taken as statistically significant; Holm–Bonferroni correction was applied to pairwise AUC comparisons.

## Supplementary Figure


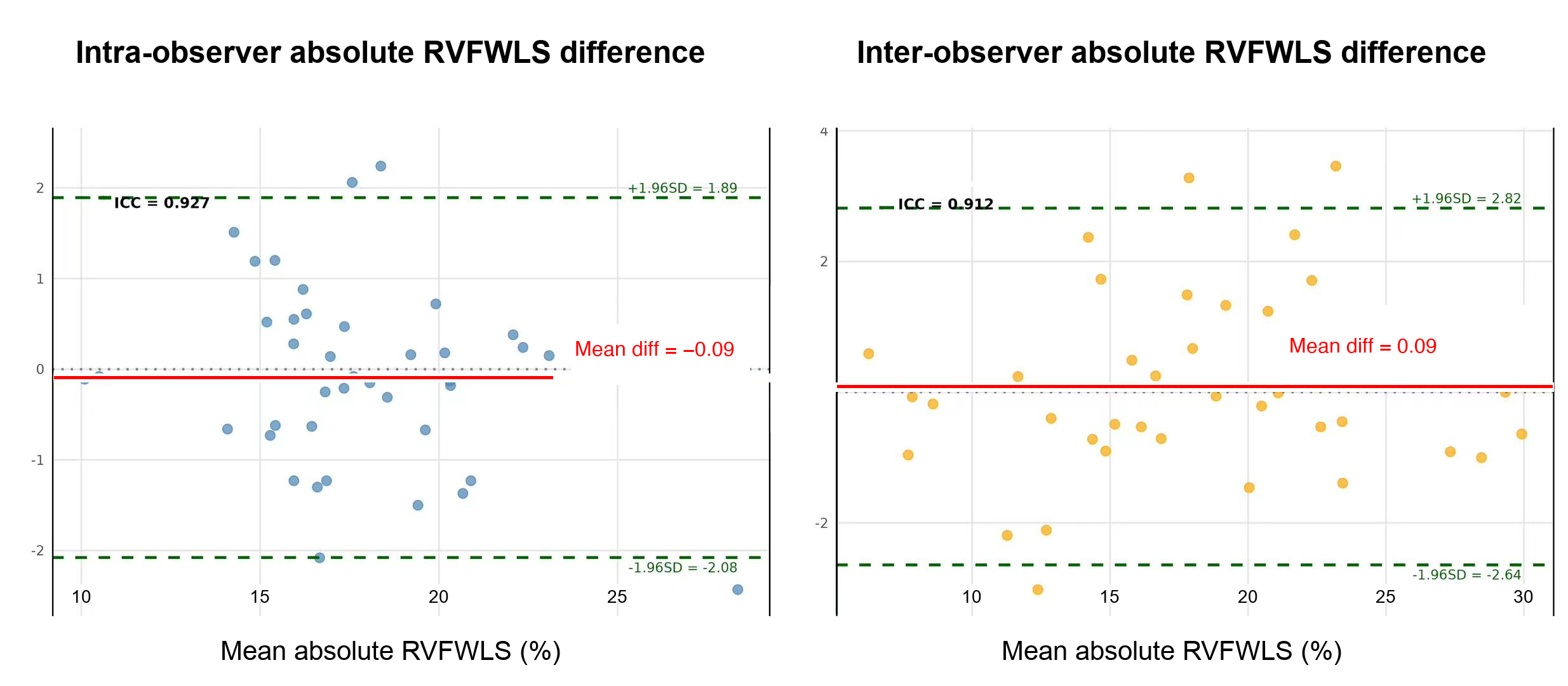


Supplementary Figure S1. Bland–Altman plots for absolute RVFWLS in the reproducibility subsample (n = 30). Left panel: intra-observer differences between the two readings by reader 1 (≥ 2-week interval). Right panel: inter-observer differences between reader 1 and reader 2. The red solid line indicates the mean difference (bias); the green dashed lines indicate the 95% limits of agreement (mean ± 1.96 SD). ICC values are annotated on each panel.


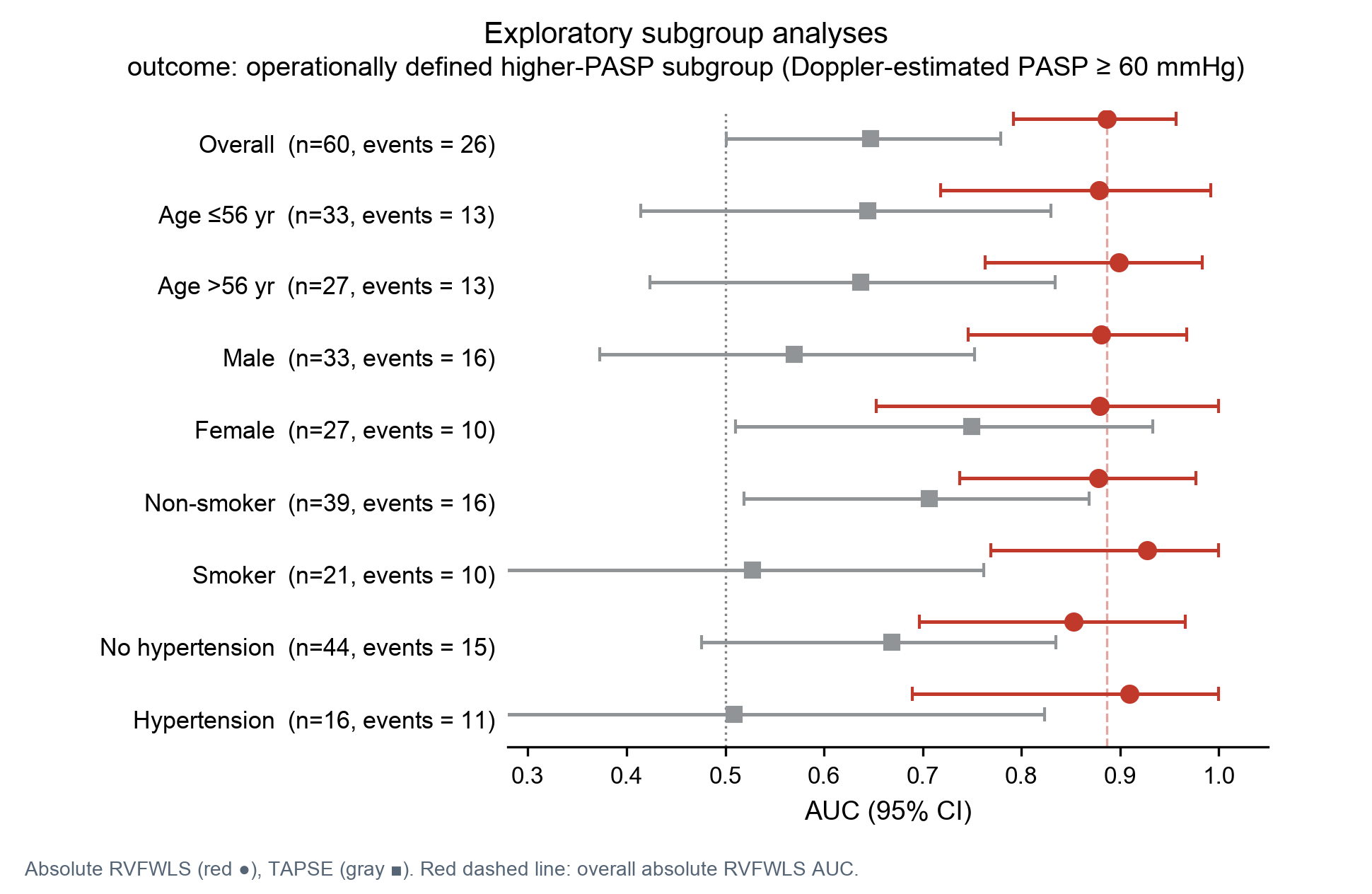


Supplementary Figure S2. Subgroup forest plot of AUCs for absolute RVFWLS and TAPSE for the operationally defined higher-PASP subgroup within HAHD. Bootstrap 95% confidence intervals were computed from 500 resamples. The vertical red dashed line marks the overall absolute RVFWLS AUC (0.886). Subgroups containing fewer than three events or three non-events were not analyzed.


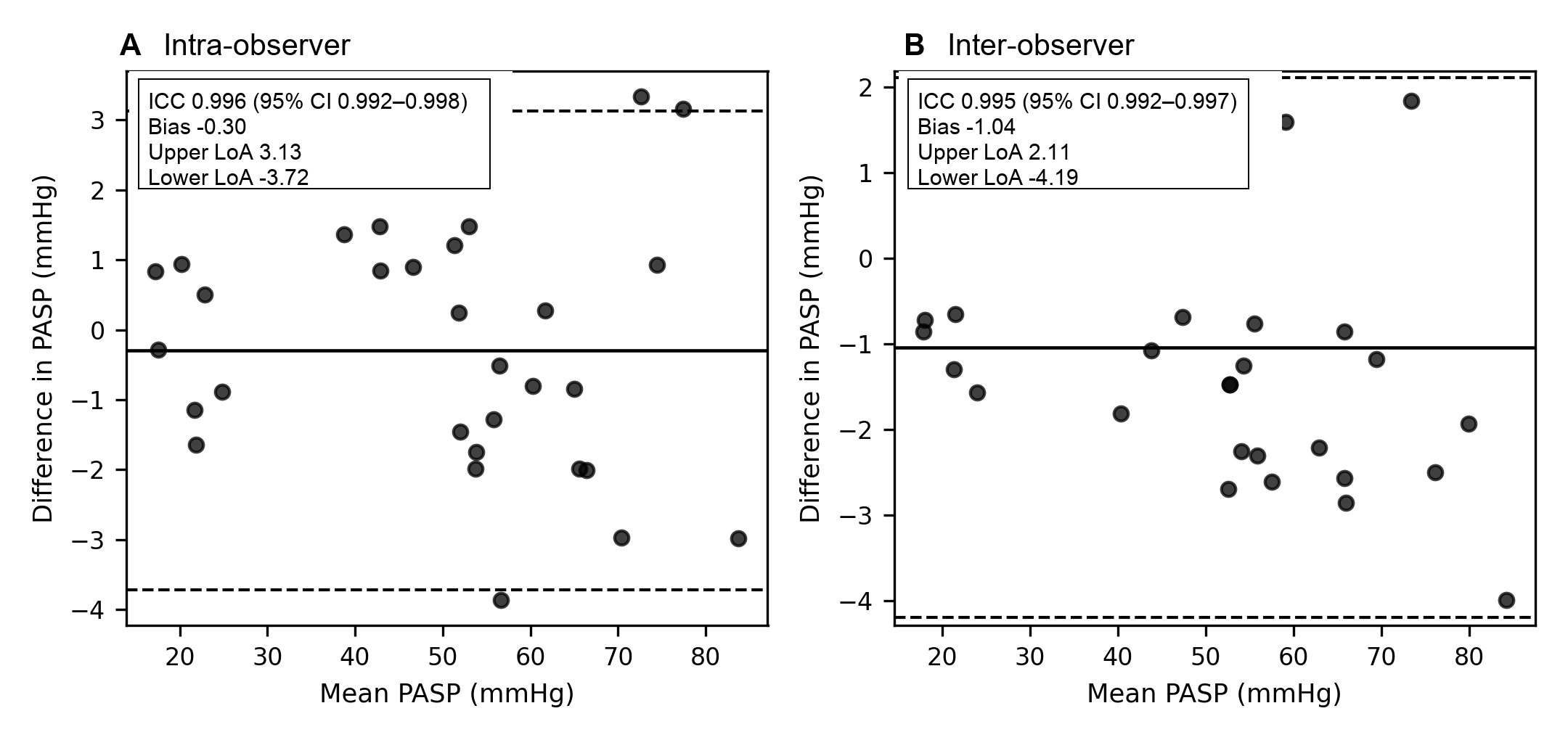


Supplementary Figure S3. Bland–Altman plots for PASP estimation in the reproducibility subsample (n = 30). Panel A shows intra-observer agreement between the two readings by reader 1. Panel B shows inter-observer agreement between the first reading by reader 1 and the independent reading by reader 2. The solid line indicates the mean difference (bias), and the dashed lines indicate the 95% limits of agreement. ICC values with 95% CIs are shown within each panel.


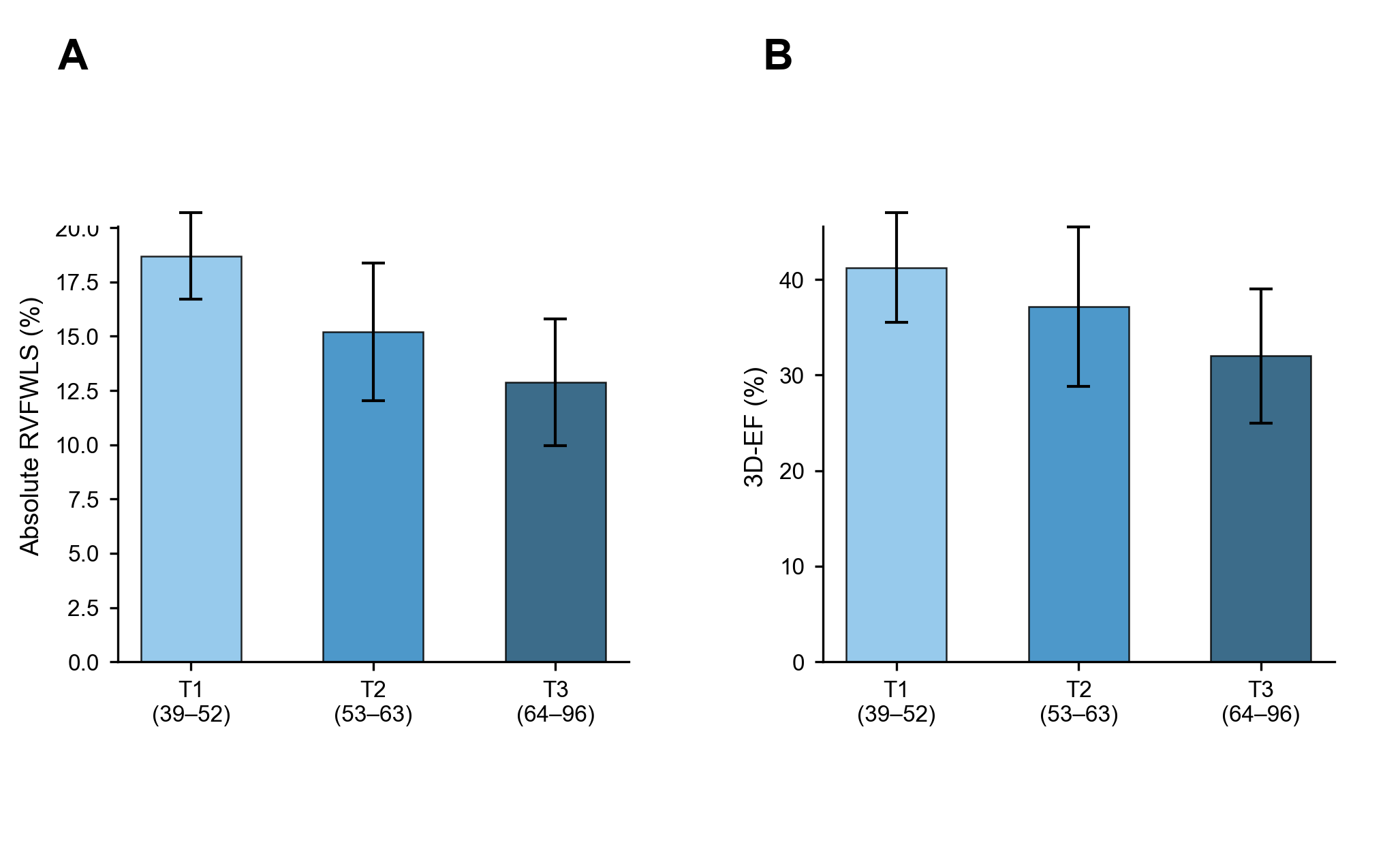


Supplementary Figure S4. Descriptive cross-sectional comparisons across PASP-defined tertiles. Panel A shows absolute RVFWLS across Doppler-estimated PASP-defined tertiles, and Panel B shows 3D-EF across the same tertiles. Because PASP was used as the grouping variable, these comparisons are descriptive only and should not be interpreted as evidence of a temporal severity gradient, disease progression, or a mechanistic relationship. PASP, pulmonary artery systolic pressure; RVFWLS, right ventricular free-wall longitudinal strain; 3D-EF, three-dimensional right ventricular ejection fraction.


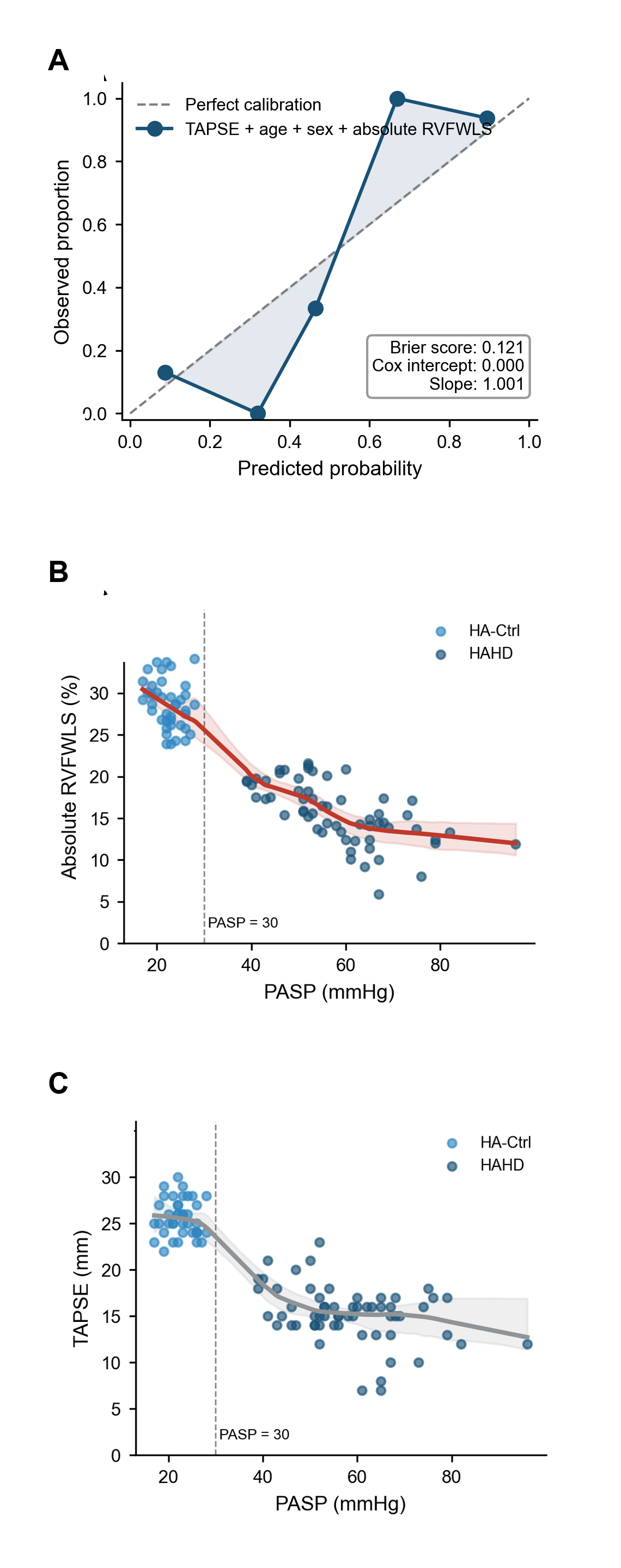

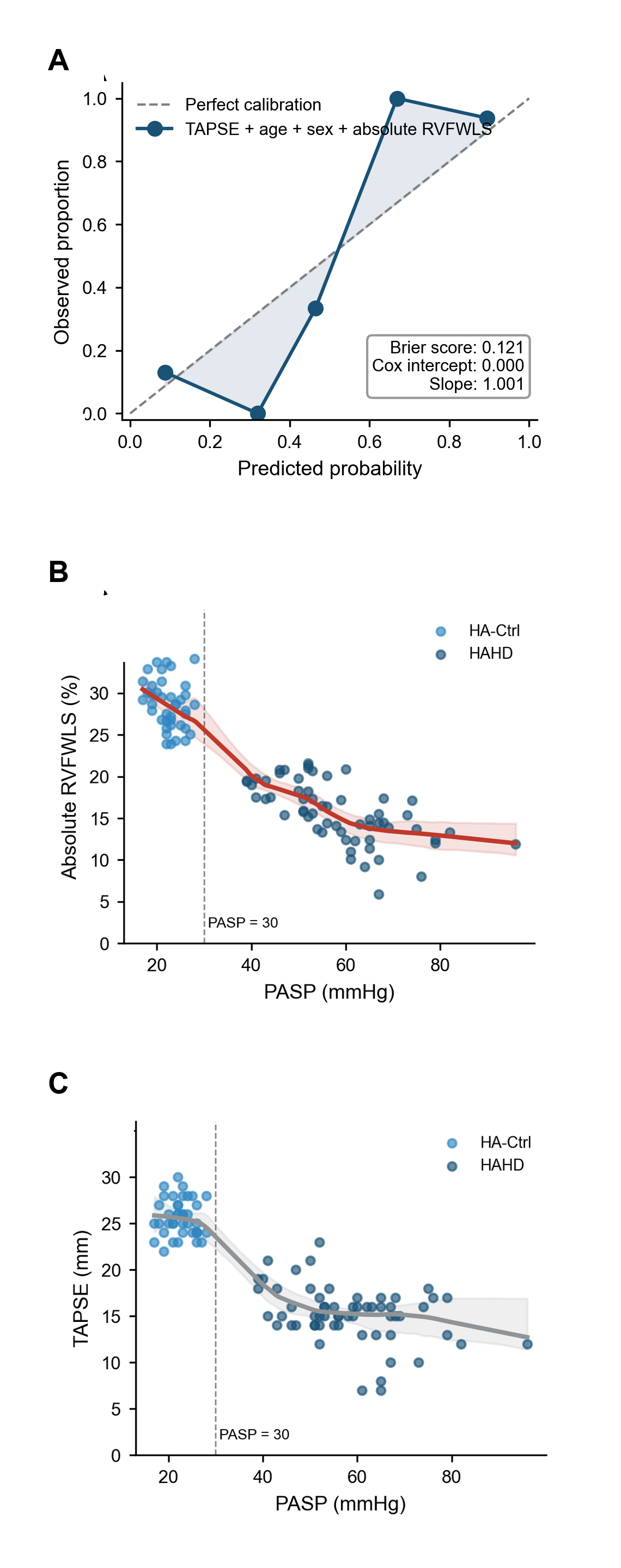


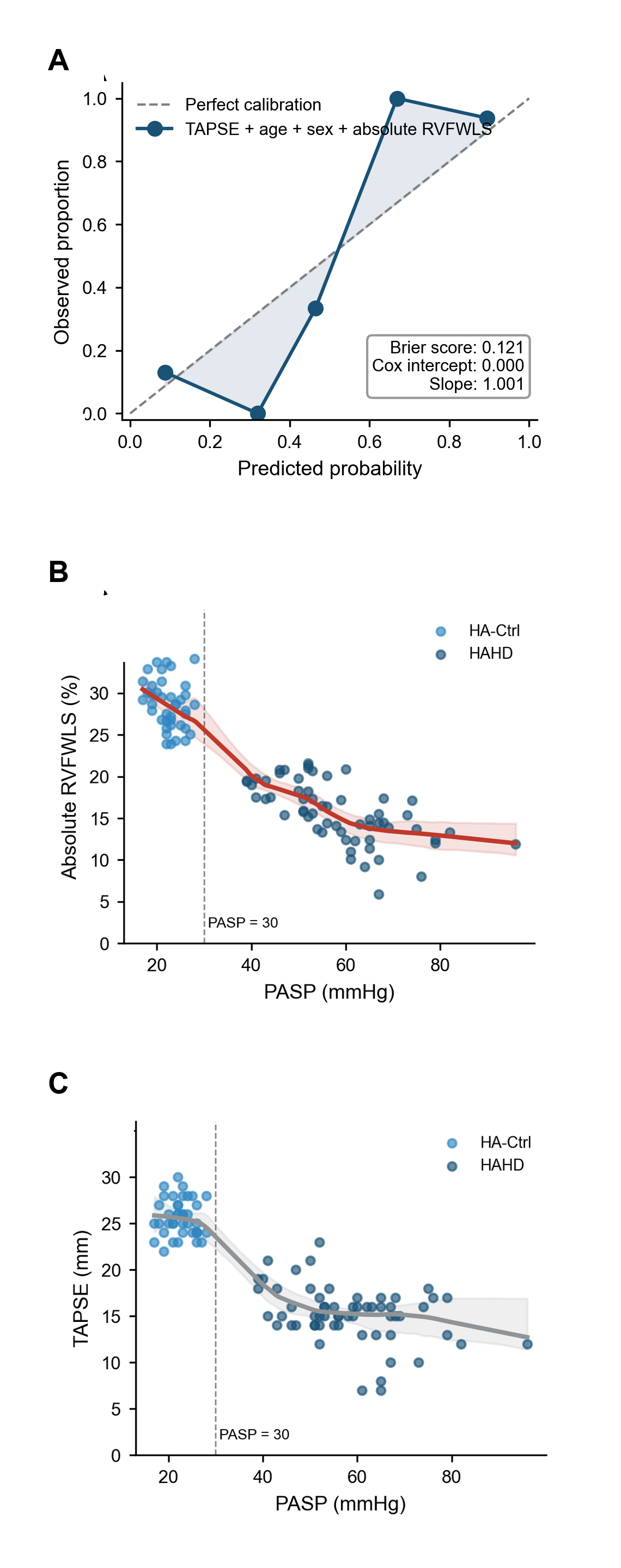


Supplementary Figure S5. Internal model diagnostics and descriptive PASP-coupling relationships. Panel A shows the apparent calibration plot for the incremental logistic model containing TAPSE, age, sex, and absolute RVFWLS. Internal validation was performed using bootstrap optimism correction. Panels B and C show descriptive scatter plots with smoothing splines for absolute RVFWLS and TAPSE against PASP; these cross-sectional plots are descriptive only and should not be interpreted as evidence of a temporal severity gradient, disease progression, or a mechanistic relationship. PASP, pulmonary artery systolic pressure; RVFWLS, right ventricular free-wall longitudinal strain; TAPSE, tricuspid annular plane systolic excursion.
