# Supplementary figures and images for: Three-dimensional right ventricular free-wall strain for identifying a higher Doppler-estimated PASP subgroup in high-altitude heart disease
Source: Front Cardiovasc Med. 2026 Jul 9;13:1885692. doi: 10.3389/fcvm.2026.1885692 (PMC13394275; doi:10.3389/fcvm.2026.1885692)

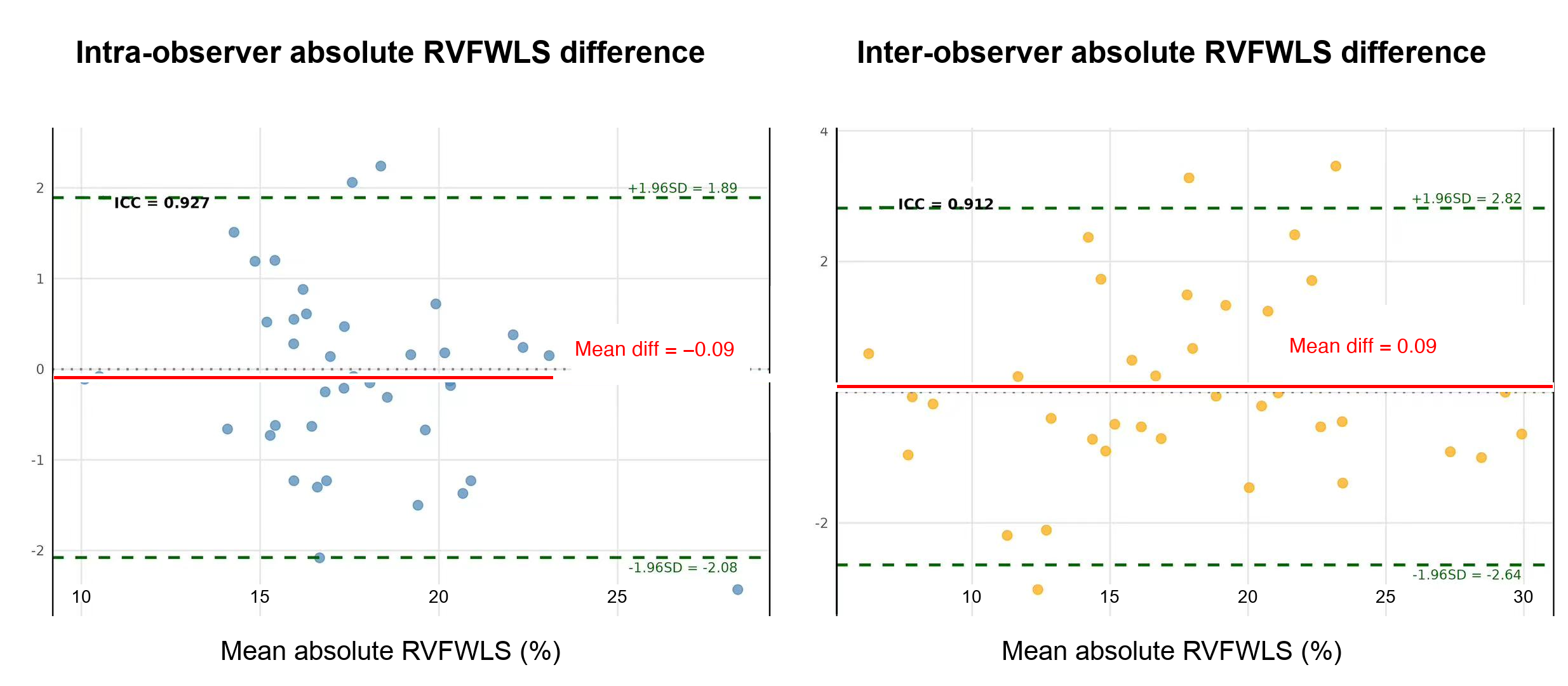

Supplement: Supplementary file 2 [file Image1.tiff]

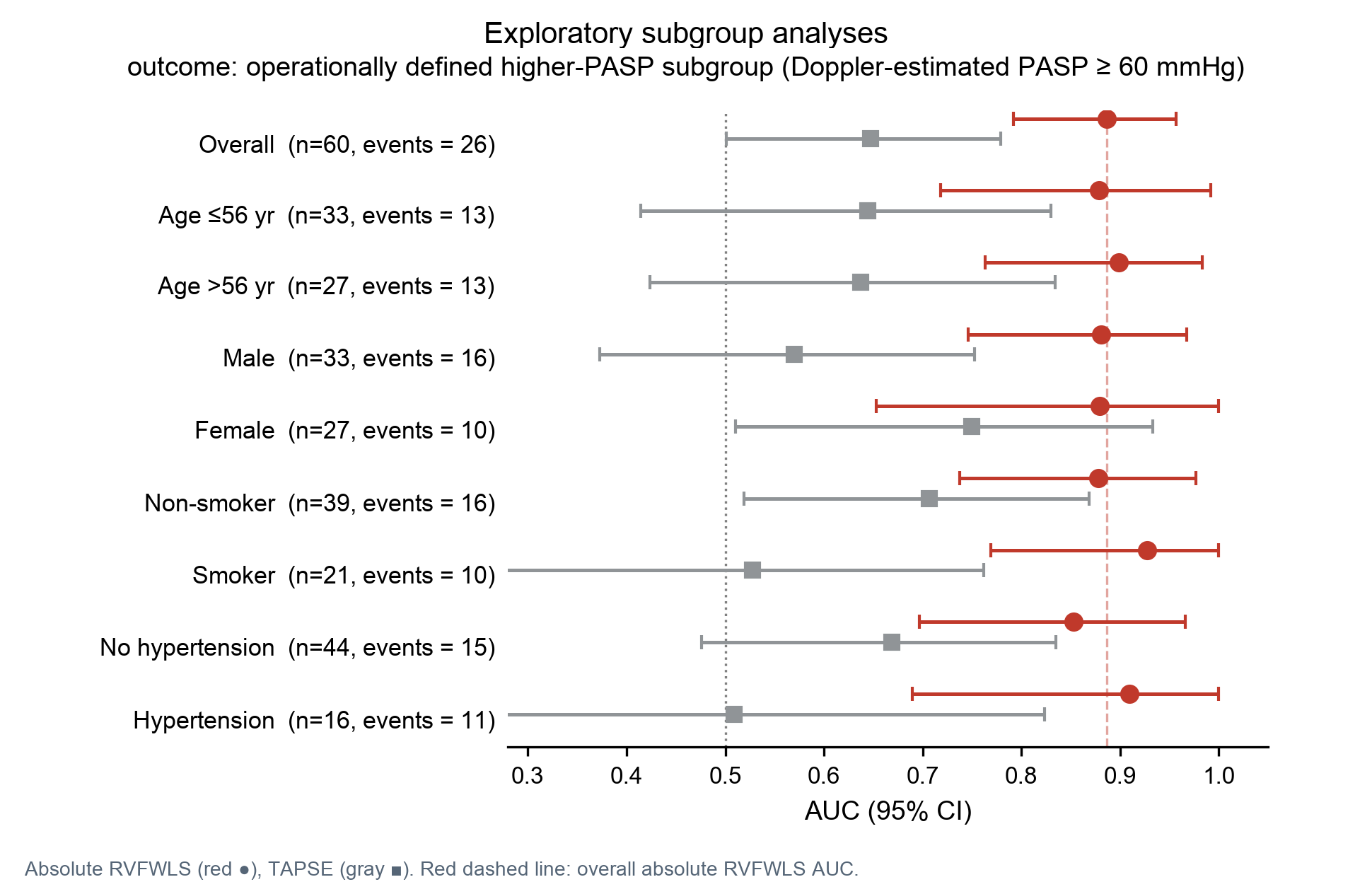

Supplement: Supplementary file 3 [file Image2.tiff]

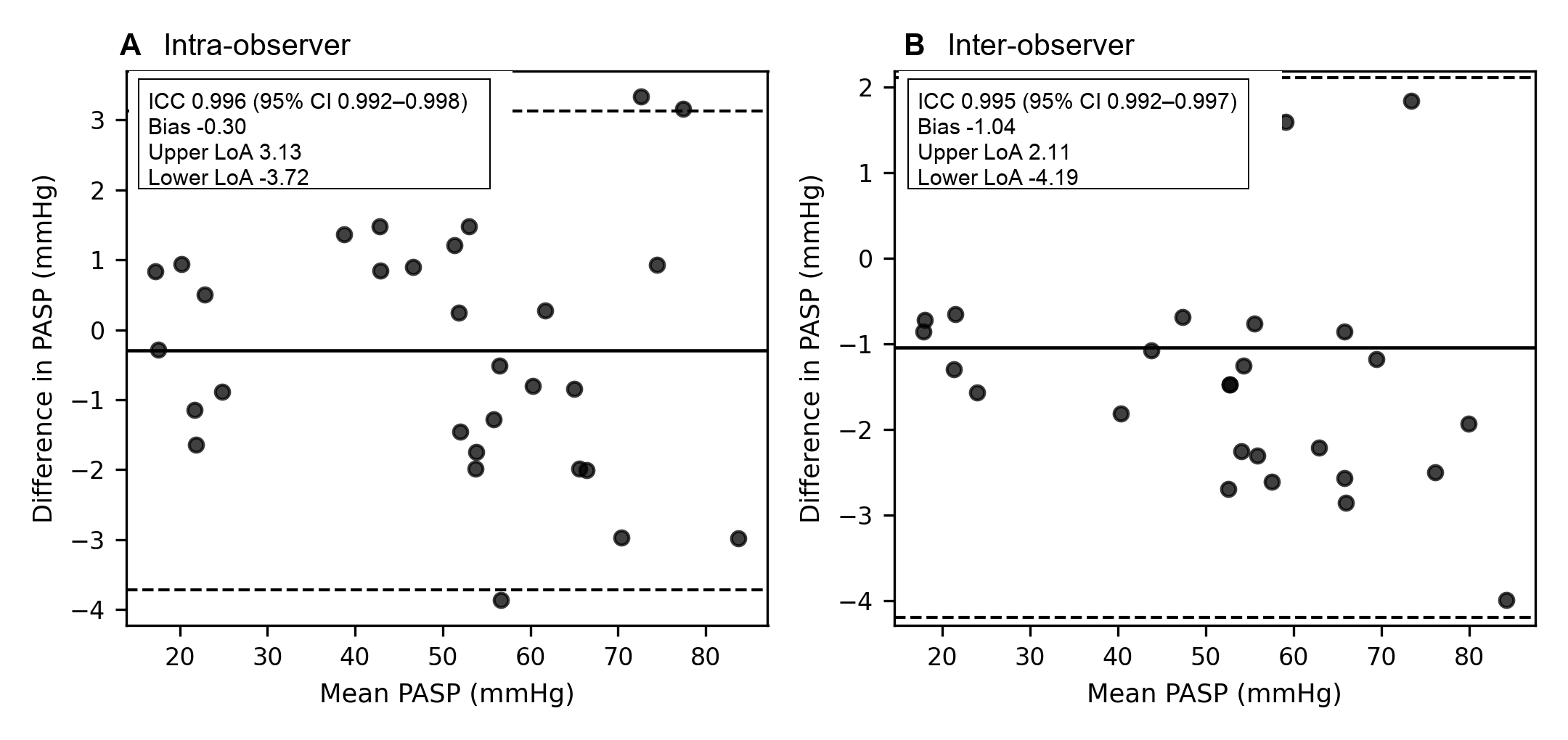

Supplement: Supplementary file 4 [file Image3.tiff]

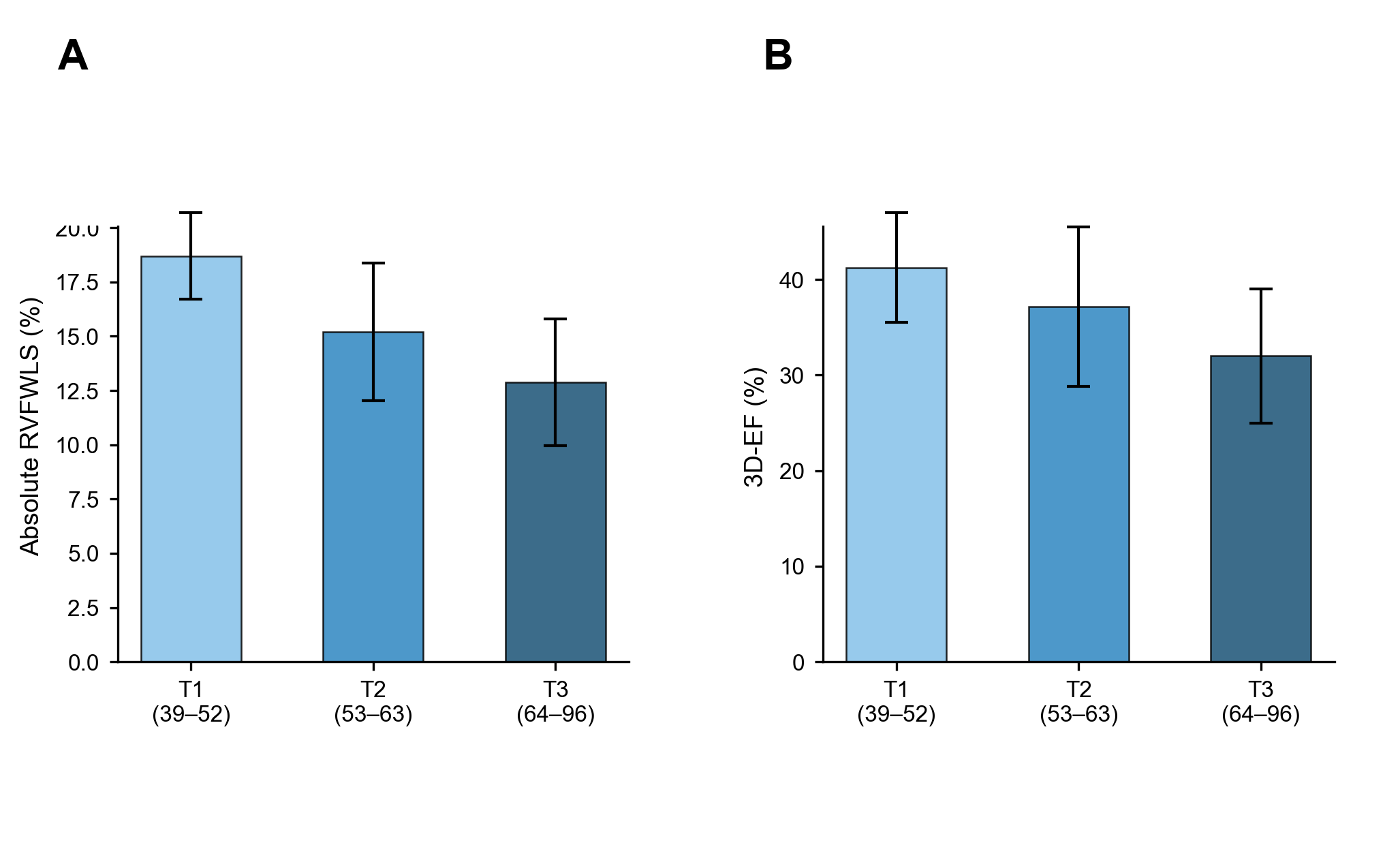

Supplement: Supplementary file 5 [file Image4.tiff]

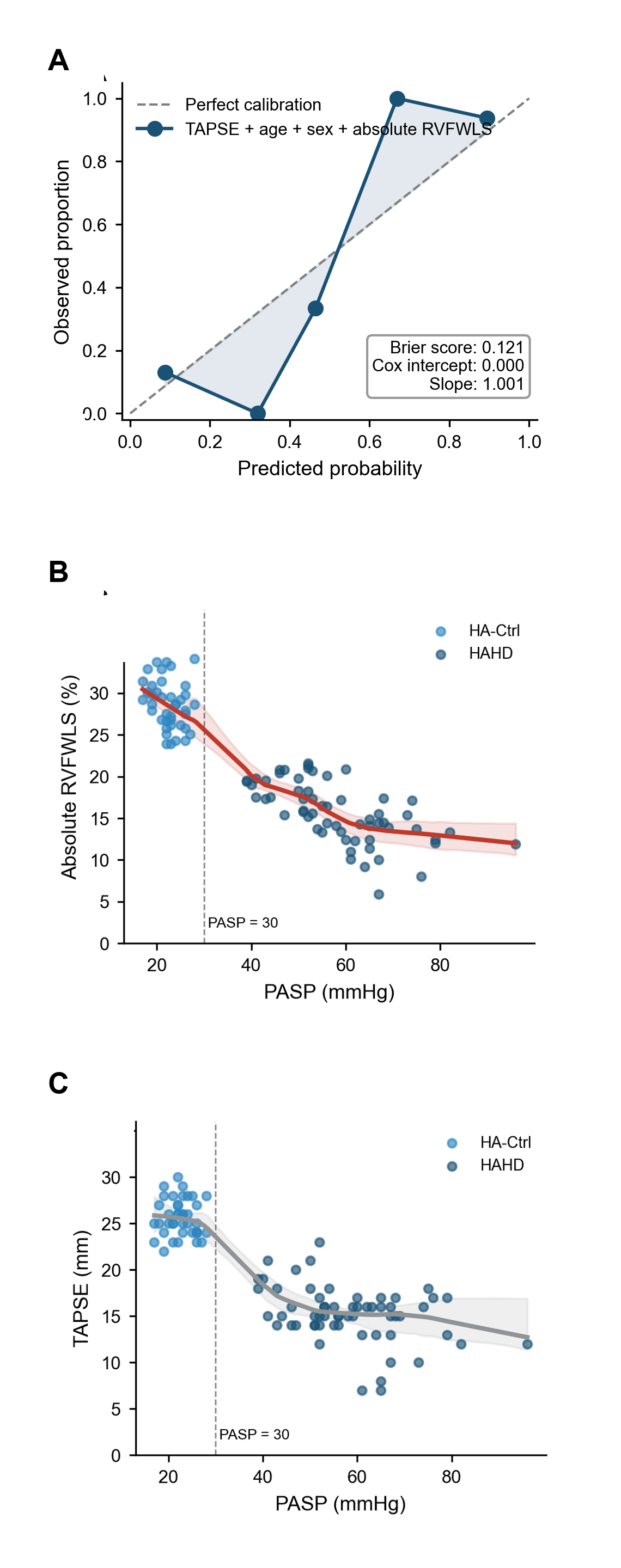

Supplement: Supplementary file 6 [file Image5.tiff]
